# The Role of Literal Features During Processing of Novel Verbal Metaphors

**DOI:** 10.3389/fpsyg.2020.556624

**Published:** 2021-01-26

**Authors:** Camilo R. Ronderos, Ernesto Guerra, Pia Knoeferle

**Affiliations:** ^1^Institut für Deutsche Sprache und Linguistik, Humboldt-Universität zu Berlin, Berlin, Germany; ^2^Center for Advanced Research in Education, Institute of Education, Universidad de Chile, Santiago, Chile; ^3^Einstein Center for Neurosciences Berlin, Berlin, Germany; ^4^Berlin School of Mind and Brain, Berlin, Germany

**Keywords:** verbal metaphors, eye-tracking, experimental pragmatics, figurative language comprehension, metaphor processing

## Abstract

When a word is used metaphorically (for example “walrus” in the sentence “The president is a walrus”), some features of that word's meaning (“very fat,” “slow-moving”) are carried across to the metaphoric interpretation while other features (“has large tusks,” “lives near the north pole”) are not. What happens to these features that relate only to the literal meaning during processing of novel metaphors? In four experiments, the present study examined the role of the feature of physical containment during processing of verbs of physical containment. That feature is used metaphorically to signify difficulty, such as “fenced in” in the sentence “the journalist's opinion was fenced in after the change in regime.” Results of a lexical decision task showed that video clips displaying a ball being trapped by a box facilitated comprehension of verbs of physical containment when the words were presented in isolation. However, when the verbs were embedded in sentences that rendered their interpretation metaphorical in a novel way, no such facilitation was found, as evidenced by two eye-tracking reading studies. We interpret this as suggesting that features that are critical for understanding the encoded meaning of verbs but are not part of the novel metaphoric interpretation are ignored during the construction of metaphorical meaning. Results and limitations of the paradigm are discussed in relation to previous findings in the literature both on metaphor comprehension and on the interaction between language comprehension and the visual world.

## 1. Introduction

In conversation, speakers usually use words in a way that is close to the word's conventional meaning. When this is the case, listeners are assumed to retrieve this word from their mental lexicon in order to grasp the meaning intended by the speaker. But what happens in a listener's mind when words are used in a previously unheard sense that requires a rapid integration of context in order to be understood? Such is the case of novel metaphors:

*1. It was difficult for the journalist to see his opinion fenced-in after the change in regime*.

In (1), a verb of physical confinement (*fenced-in*) is used to predicate over an abstract noun which does not have a physical dimension (*the journalist's opinion*), yet the intended meaning can be readily derived: The journalist is no longer allowed to speak freely. In this example, the feature of “physical confinement” is not part of the metaphor and is even incompatible with the speaker's intended meaning.

The role ascribed to features that relate only to the literal meaning of a word and are incompatible with that word's novel metaphorical meaning (henceforth “literal features”) during processing varies depending on the theoretical perspective (see Holyoak and Stamenković, 2018 for a systematic review of competing views). Some accounts see metaphor comprehension as a type of category inclusion: They claim that understanding a metaphor, such as *The president is a walrus* involves a contextual adjustment of the meaning of the metaphoric vehicle (*walrus*) on the basis of the dimensions provided by the metaphoric topic (*The president*). Language comprehenders thus create a new, occasion-specific category (McGlone and Manfredi, 2001; Glucksberg, 2003; Rubio Fernandez, 2007; Sperber and Wilson, 2008), an idea inspired by Barsalou's work on *ad hoc* categories (Barsalou, 1983). This type of meaning modulation unfolds via rapid suppression of incompatible literal features (e.g., “has large tusks,” “lives near the north pole”) and enhanced activation of only those features that are compatible with the dimensions provided by the metaphoric topic and are relevant for interpretation (e.g., “very fat,” “slow-moving”) (Gernsbacher et al., 2001).

A competing set of views sees metaphor understanding as a process of indirect comparison. When encountering a metaphor, we reason analogically about the conceptual structure of both topic and vehicle in order to reach a final utterance interpretation (Gentner and Holyoak, 1997; Wolff and Gentner, 2000, 2011; Coulson and Oakley, 2005; Gentner and Bowdle, 2008). A necessary first step in this process is that of structural alignment: Topic and vehicle are scanned for commonalities in their structures, and only after these commonalities have been established, inferences are projected from vehicle to topic. Here, metaphor-incompatible features of the vehicle are not immediately suppressed and can only be discarded after structural alignment has been achieved (McGlone and Manfredi, 2001). The “career of metaphor” hypothesis (Bowdle and Gentner, 2005), an extension of the indirect comparison view, claims that there is a difference in processing between novel and conventional metaphors. For conventional metaphors, they claim that meaning is not constructed via analogical reasoning but is instead retrieved via category selection. Researchers working within the framework of category inclusion, however, have argued against this providing evidence suggesting that not conventionality but aptness (i.e., how “good” a metaphor is) determines a metaphor's processing mode, meaning that there should not be an a priori difference in processing route between novel and conventional metaphors (Jones and Estes, 2006)^1^.

Several studies have dealt with whether these literal features are activated or suppressed during processing (and if so, when). As a whole, the results do not unequivocally support one or the other set of accounts (e.g., Gernsbacher et al., 2001; McGlone and Manfredi, 2001; Rubio Fernandez, 2007; Weiland et al., 2014). We argue that three common features of these studies could be improved upon when striving for consensus. Firstly, these studies restricted their investigations to sentences, such as “Some lawyers are sharks” (known in the literature as nominal metaphors), in which both metaphoric topic and vehicle are nouns and they have the surface form of a category statement. Considering that metaphors in the wild can take a wide range of morphosyntactic forms (see for example Bambini et al., 2019), it is problematic for theory development to consider only a small subset of metaphors.

Secondly, these studies usually make use of materials in which the relation between the metaphors and the tested literal features varies for every item. For example, two of the metaphoric items from McGlone and Manfredi (2001) (one of the most prominent studies on the role of literal features during metaphor comprehension) were *some stomachs are barrels* and *some cats are princesses*. The study examined the relationship between these sentences and the literal features captured in the sentences *barrels can be wooden* and *cats can be siamese*, respectively. *Wooden* and *siamese* are very different types of properties that require different kinds of world knowledge from a listener, and it is unclear to what extent we can meaningfully compare the relationship of each of these literal sentences to its metaphoric counterpart. It could be the case that variation in the relationship between literal features and target metaphors across experimental items is (at least partially) responsible for some of the contradictory results in the literature. A similar argument was made by Thibodeau and Durgin (2008) with regards to the difference in results of their study (facilitation effect of conventional metaphors on processing subsequent related novel metaphors) when compared to the results of Keysar et al. (2000) (no facilitation effect of conventional metaphors on processing subsequent related novel metaphors).

Finally, the majority of experiments investigating the role of literal features of a metaphor have been conducted using sentence reading times or reaction times as the dependent measures (but see Weiland et al., 2014, for a notable exception). As a result, the timing of the activation of literal feature representations remains unclear and should be addressed with a finer-grained method. With the present set of studies we intend to make a contribution to the debate on the role of literal features during metaphor processing by improving on these three issues.

Concretely, we set out to study the role of conceptual features that are part of the encoded meaning of a verb but are incompatible with its novel metaphoric interpretation: We conducted a series of experiments investigating the role of the specific feature of physical containment during processing of novel verbal metaphors, such as (1). In these metaphors, the vehicle is always a verb of physical containment used to signify difficulty. This allowed us to use the same animated videos displaying physical containment as a visual representation of the same literal feature across items. We based our paradigm and hypotheses on insights coming from psycholinguistic accounts of metaphor comprehension (Glucksberg, 2003; Gentner and Bowdle, 2008), as well as from research on metaphor production (Sato et al., 2015). Crucially, we relied on the insights and on the methodology of research conducted on the interaction of (written) language processing and the visual context (Guerra and Knoeferle, 2014, 2017) to create our experimental paradigm.

The paper is structured as follows: The next two subsections provide an overview of the different views on metaphor processing and their predictions and briefly introduce the literature on the interaction between language processing and the visual world. We then present two eye-tracking during reading studies, one self-paced reading experiment and one lexical decision task, all investigating to what extent a depiction of physical containment influences the processing of novel verbal metaphors. Results are discussed in light of the background presented in section 1.

### 1.1. Understanding Metaphors

An issue of importance for metaphor theories is the role of the literal meaning of a metaphoric vehicle during processing: In (1), the verb *fenced-in* entails the concept of physical containment; its direct object is something that is not allowed to physically move. However, when we hear that *the journalist's opinion has been fenced-in*, the feature of a physical barrier is not part of the final interpretation. What happens to this **literal feature** during comprehension?

From a category inclusion perspective, the noun *opinion* in (1) provides the dimension of [+ abstract]. This dimension, together with the relevant utterance context, determines the interpretation of the verb: relevant features are selected while irrelevant ones are actively discarded. Evidence for this view comes from priming experiments. Gernsbacher et al. (2001) showed participants either a metaphoric or a literal sentence as a prime (*That defense lawyer is a shark* or *That large hammerhead is a shark*) and then asked them to perform a verification task on a sentence describing a feature of the vehicle that was irrelevant or relevant for the construction of the metaphoric meaning (*sharks are good swimmers* or *sharks are tenacious*). They found that, after reading metaphorical primes, participants were faster at verifying sentences describing a relevant feature for the metaphoric interpretation compared to when they read a literal prime. They also found that verifying sentences about a metaphor-irrelevant property took longer after reading a metaphor than after reading a literal statement. They interpreted these results in terms of activation of relevant features and suppression of irrelevant ones: When the word *shark* is used metaphorically, features, such as “tenacious” are enhanced and features, such as “good swimmer” are inhibited.

Rubio Fernandez (2007) conducted a similar study with the key difference that the target was a single word and it was shown at varying intervals. She found that at early intervals (0 and 400 ms) irrelevant literal features were primed by the metaphor and only actively suppressed when presented 1,000 ms after the prime. McGlone and Manfredi (2001) deployed a reversed version of this paradigm and showed participants irrelevant or relevant features as primes and then metaphorical sentences as targets. They found that relevant features facilitated whereas irrelevant features hindered comprehension compared to a baseline condition without a prime, suggesting that irrelevant properties are suppressed early on during processing. Weiland et al. (2014) created an ERP version of this paradigm: they showed participants a masked prime consisting of a word representing an irrelevant feature (*furry*) of a metaphor (*my lawyer is a hyena*) followed by the metaphor itself. They found that the N400 effect (computed as the difference in stimulus-related average electrical responses between the metaphor and a literal equivalent) was reduced when participants saw the irrelevant prime compared to when they did not see any prime at all, suggesting that irrelevant features can indeed ease comprehension of a metaphor, a result which is in conflict with that of McGlone and Manfredi (2001).

From the perspective of indirect comparison, on the other hand, the activation of relevant and irrelevant features of the vehicle are not contingent upon dimensions provided by the topic. Gentner and Holyoak (1997), Gentner et al. (2001), Bowdle and Gentner (2005), Gentner and Bowdle (2008) have argued that, during initial stages of comprehension, the elements of a novel metaphor are scanned for structural similarities: listeners reason analogically about the relationship between vehicle and topic. This requires irrelevant features of the vehicle to be initially activated and only suppressed or ignored during later stages, once structural alignment has already taken place (Gentner and Bowdle, 2008). This view is compatible with the findings of Weiland et al. (2014) but incompatible with those of McGlone and Manfredi (2001). According to the indirect comparison view, it is also likely that literal features remain active after a metaphor has been understood, because the pattern of structural mappings between topic and vehicle can be used for subsequent processing, as has been shown to be the case for extended metaphors. For these, words belonging to the same semantic domain are used to “extend” a metaphoric expression beyond a single topic-vehicle pairing, as in the famous lines from Shakespeare's *As you like it*: “All the world's a stage and all the men and women merely players; they have their exits and their entrances, and one man in his time plays many parts.” Support for this view comes from priming paradigms, where it has been shown that novel metaphors facilitate processing of subsequent novel metaphors that share the same conceptual mappings between domains (Keysar et al., 2000) and even that conventional metaphors can prime subsequent related novel metaphors (Thibodeau and Durgin, 2008).

Findings on extended metaphors are somewhat challenging to account for from the perspective of category inclusion, which seems to posit that metaphor comprehension occurs only locally: If the meaning of the metaphoric vehicle is altered so that irrelevant literal features are suppressed, how can these features be re-activated to prime subsequent related metaphors? One answer, coming from within Relevance Theory, is given by Carston (2010). She claims that, in an extended metaphor, the multiple related words that are semantically associated are mutually reinforcing, resulting in an enhanced activation of the literal meaning (which she calls the “lingering” of the literal meaning). This can lead to the entire literal meaning of the extended metaphor to be meta-represented and considered as a sort of “imaginary world,” where the individual metaphors are understood literally. This activates a second processing route for extended metaphors where metaphoric meaning is only derived in later stages of processing (Rubio-Fernández et al., 2016).

Regarding the activation of literal features, the difference between indirect comparison views and Carston (2010) seems to be that Carston (2010) might predict a facilitation effect of metaphors on subsequent related metaphors based on semantic reinforcement of related words, whereas Gentner et al. (2001) predicts a general activation of structural mapping patterns after any metaphor has been activated. In other words, the indirect comparison view predicts that literal features of a metaphor remain active after a metaphor is understood because these are part of a complex network of mappings between the encoded meanings of the metaphoric topic and the metaphoric vehicle. The category inclusion view of Carston (2010), on the other hand, predicts activation of the encoded semantic features of a metaphoric vehicle (i.e., “lingering” of the literal meaning), not of a network of systematic mappings.

In short, there is a lack of consensus in the literature on the timing of suppression and activation of literal features during and after metaphor comprehension: whereas, it has been suggested that irrelevant literal features hinder processing (McGlone and Manfredi, 2001) and are immediately suppressed after comprehension (Gernsbacher et al., 2001), others claim that literal features can ease subsequent processing of a metaphor (Weiland et al., 2014), remain active for at least 400 ms after processing (Rubio Fernandez, 2007), and even facilitate processing of subsequent related metaphors (Thibodeau and Durgin, 2008). It is therefore important to seek out more evidence in this debate since it has repercussions for theory development, as highlighted above.

It is crucial to note that the activation of literal features during metaphor comprehension could be affected by item-specific factors, such as a metaphor's conventionality (i.e., the subjective frequency of exposure to a specific vehicle in its metaphoric meaning) (e.g., Blasko and Connine, 1993; Wolff and Gentner, 2000; Bowdle and Gentner, 2005). It could, for instance, be the case that literal features facilitate access for novel metaphors and hinder comprehension for more conventional metaphors (in line with the “career of metaphor” hypothesis, Bowdle and Gentner, 2005). McGlone and Manfredi (2001), for example, found that the metaphors in their study that were rated as less conventional displayed less interference from irrelevant literal features during processing compared to the metaphors that were rated as more conventional. The effect was nevertheless one of interference, for both novel and conventional metaphors separately (−8 and −143 ms, respectively) and when taken as a whole. Weiland et al. (2014), on the other hand, controlled for conventionality by selecting only metaphors that were rated as being halfway between highly novel and highly conventional for their experiments, and did not report any mediating effect of conventionality. Gernsbacher et al. (2001) operationalized conventionality as the percentage of comprehension errors for each of the metaphors in their study. They found that it did not correlate with the effect size of each of the items in their experiment, suggesting that conventionality did not modulate the way that literal features were suppressed after metaphor comprehension. Finally, Rubio Fernandez (2007) did not report having controlled for conventionality. This specific literature therefore does not strongly suggest that conventionality mediates the role of literal features during metaphor comprehension. Furthermore, it is still an open question whether conventionality actually modulates processing, or whether it only appears to do so because it tends to be correlated with aptness (i.e., the degree to which the figurative meaning of the vehicle captures relevant properties of the topic, or how “good” the metaphor is), which has been claimed by some to be the true underlying factor that mediates metaphor processing (Jones and Estes, 2006; Glucksberg, 2008). An investigation on the effect of conventionality on the activation of literal features is beyond the scope of our current investigation, which focuses on the processing of novel metaphors exclusively.

Specifically, our contribution to the debate on the activation of literal features is to examine the effect of pre-activating said features on the processing of subsequent verbal metaphors, which, unlike nominal metaphors, have been largely overlooked in the literature. We do this by showing participants short animated clips of the literal feature of containment prior to participants reading verbal metaphors that entail this feature as part of their literal meaning. Our study makes use of eye-tracking during reading and draws its inspiration from research on the relation between visual attention and language production and processing. We will now turn to a brief overview of this specific research field.

### 1.2. The Interaction of Visual and Linguistic Information During Sentence Processing

Given the lack of converging evidence coming from the studies described above, we turned to neighboring disciplines for inspiration. One possibility is to draw from research on language-vision interactions (see Knoeferle and Guerra, 2016 for an introduction to this field). The seminal work of Cooper (1974) showed that there is a close temporal adjacency between language understanding and the processing of visual stimuli. In the study, participants heard stories while simultaneously being presented with images of potential referents while their eye movements were monitored, something that years later came to be known as the Visual World Paradigm (for a review, see Huettig et al., 2011). The results of this study showed that participants looked at the visual representations of objects immediately after they were mentioned in a story, highlighting the rapid and automatic way in which language and visual processes interact.

Through eye-tracking technology it has also been shown that the processing of visual stimuli interacts with the processing of written abstract language. Guerra and Knoeferle (2014) showed participants a video of two playing cards that either moved closer together or further apart. Participants then read German sentences that dealt with semantic dissimilarity, such as *Frieden und Krieg sind bestimmt verschieden* (“Peace and war are certainly different”) or similarity, such as *Kampf und Krieg sind freilich entsprechend* (“Battle and war are certainly similar”). Their results showed that when the motion of the cards was conceptually aligned with the direction of the semantic relation (close~similar; far~different), participants were faster at reading the second of the presented nouns (Experiment 3) as well as the adjective (Experiments 1 and 2) than when there was no such conceptual alignment. The result was interpreted as evidence for an abstract co-indexing link between spatial distance and semantic similarity. One characteristic of the eye-tracking during reading method is that it allows for a rough mapping of the results onto different stages of language processing (Clifton et al., 2007; see Vasishth et al., 2013, for a counterpoint). The fact that Guerra and Knoeferle (2014) found effects in first-pass reading times (considered a measure of early stages of processing) can be interpreted as a sign of the early and rapid integration of language processing and the visual context.

It's important to note that Guerra and Knoeferle (2014) investigated the effects of the visual context on the processing of concepts that have been retrieved from memory, such as the meaning of the words “war” and “peace.” But how does the visual world interact with processing concepts that are not retrieved from one's mental lexicon, but are instead constructed on the fly, such as novel metaphors? We might find an answer to this question if we look at how the visual world interacts with the production of metaphoric expressions. Sato et al. (2015) investigated whether showing participants images depicting spatial containment would encourage them to produce expressions in which spatial containment is used metaphorically to speak of abstract difficulty. They found that even when the sentences they produced were thematically unrelated to the images viewed, participants still produced more metaphors drawing from the domain of spatial containment than when they saw a neutral picture as prime. The authors, who work within the framework of Conceptual Metaphor Theory (Lakoff and Johnson, 2008), interpreted the result as evidence for an activation of the Conceptual Metaphor DIFFICULTY IS CONTAINMENT after having seen the pictures, leading to the production of individual linguistic metaphors derived from this specific Conceptual Metaphor.

It's possible that these results could translate to language comprehension: activating the feature of spatial containment could facilitate comprehension of novel metaphors of difficulty that have spatial containment as part of their encoded meanings. This would suggest that literal features of a metaphor are important for the construction of metaphoric meaning and would be broadly in line with the indirect comparison view. With this in mind, we now turn to the description of our investigation, in which we explore the role of literal features during comprehension of novel verbal metaphors.

## 2. The Present Study

The current set of studies seeks answers to the following questions raised in section 2: Does activating literal features hinder or ease processing of novel metaphors? And do said features remain active after a metaphor has been understood? We conducted four experiments to answer these questions. In Experiments 1 and 2 (eye-tracking during reading), participants saw short animated clips depicting physical containment. They then read sentences in which verbs of physical containment were metaphorically used to signify difficulty [such as in sentence (1)], and then answered questions about either the sentences or the videos. The animated clips showed a moving ball: In one video, the ball bounces freely while in the other the ball is trapped by a box.

The goal of these two experiments was to study how seeing a video depicting physical containment—which we assume to be a prominent feature of the encoded meaning of the verbs used in all our sentences, yet incompatible with the meaning of the individual metaphors—interacts with the processing of verbs of spatial containment used metaphorically. We compared this to how the same sentences are processed after seeing a video clip that does not share the conceptual feature of containment with the verbs. In these two experiments participants also answered questions about what they saw in the video after reading the sentence. This should provide insight on the role that literal features might play after a metaphor has been understood.

In Experiment 3 (self-paced reading), we examined how participants would naturally answer the same questions asked in experiments 1 and 2 (after sentence comprehension) when the video clips are followed by literal sentences instead of metaphors. Doing this gives us a baseline measure to interpret the results of the question-answering times of Experiments 1 and 2.

Finally, Experiment 4 (lexical-decision task) investigated how the same video clips of Experiments 1–3 interact with the processing of spatial containment verbs from Experiments 1 and 2 when these verbs are read in the absence of a context (i.e., when participants are expected to retrieve the literal meaning only).

## 3. Experiment 1

We began our investigation by asking the following question: Will watching video clips of spatial containment facilitate or hinder comprehension of metaphors made up by verbs of spatial containment? Additionally, how will the activation of spatial containment interact with processing the metaphorically used verbs after the metaphors have been understood? Experiment 1, an eye-tracking during reading study, was designed to answer these questions.

### 3.1. Participants

Forty-eight monolingual university students who were native speakers of German (ages 18–31, 30 female) were recruited and tested at the Humboldt-Universität zu Berlin. All participants were right handed and had normal or corrected-to-normal vision. They all gave their written informed consent and were payed 8 euros upon completing the experiment. This study was covered by the ethics vote granted to the psycholinguistics lab of the Humboldt-Universität zu Berlin by the German Linguistic Society (Deutsche Gesellschaft für Sprachwissenschaft, DGfS).

### 3.2. Materials and Design

We created 40 critical items consisting of German metaphorical sentences. All sentences had an identical syntactic structure, namely a main clause with an infinitive subject clause, as exemplified in (3). In the infinitive clause, a verb of physical containment, which always appeared in the same position, was used metaphorically to denote abstract difficulty. In the main clause, it was asserted that the situation described in the infinitive clause was “difficult.” All critical and filler sentences can be found in the Supplementary Material.

(3) *Es war für den Redakteur /schwierig* *adj* */, seine / Meinung* *target noun* */ nach dem Regimewechsel / umgittert* *verb**/ zu sehen*.
  *It was for the journalist/ difficult* *adj* */, his / opinion* *target noun* */ after the change in regime/ fenced-in* *verb* */ to see*

“It was difficult for the journalist to see his opinion be fenced-in after the change in regime.”

#### 3.2.1. Sentence Norming

Our goal when creating the materials was to use metaphors that were novel yet readily understandable. To make sure the metaphors could be understood, we conducted a norming study of the target sentences. A sample of 15 participants, who did not participate in the main study, were asked to rate 80 sentences on a scale of 1–7, with 1 being totally incomprehensible and 7 being totally comprehensible. The 80 sentences were made up of the critical 40 metaphoric sentences and 40 semantically incoherent filler sentences (e.g., *It was sad that Thomas drank the car so fast*). Order of presentation of the sentences was randomized. The goal of the norming task was to establish whether any of the critical metaphorical sentences would be rated as incomprehensible (meaning a rating of 3.5 or lower) and whether the metaphorical sentences were rated significantly higher than the semantically incoherent sentences.

#### 3.2.2. Results of the Norming Task

Four of the forty critical sentences were rated lower than 3.5 on average and were dropped from the investigation. The remaining 36 sentences formed the base for all subsequent experiments.

To determine whether these 36 sentences were in fact understood, an ordered logistic regression model was fitted to the data (Gelman and Hill, 2006). The model was constructed to see whether our critical items and the semantically incoherent fillers could predict the 1–7 ratings. The results show that a change from level 0 (semantically incoherent) to level 1 (critical item) was associated with an increase of odds ratio of 7.96 (t = 17.5, *p* < 0.001) This means that for metaphorical sentences, the odds of being rated higher were 7.96 times those of incoherent sentences, holding constant all other variables. The data therefore strongly suggests that participants were able to determine a difference in meaning between the semantically incoherent sentences and the novel metaphoric sentences.

Finally, to confirm that the resulting 36 sentences were in fact perceived as novel, we asked a further 50 participants (who did not take part in the main experiment) to rate how familiar they thought the metaphoric sentences were on a scale from 1 (very novel) to 100 (very familiar). The mean familiarity score was 27.98 with a standard deviation of 10.2. We take this as confirmation that the metaphors created were indeed perceived to be fairly novel.

#### 3.2.3. Filler Sentences

Seventy-two filler sentences were constructed to reduce the likelihood of strategic behavior and to mask the purpose of our investigation. We thus had 24 German idioms as fillers with similar syntactic structure to our critical items, as well as 24 novel metaphors different to the critical items. The remaining 24 filler sentences were literal statements.

#### 3.2.4. Visual Primes

**Figure 1:**
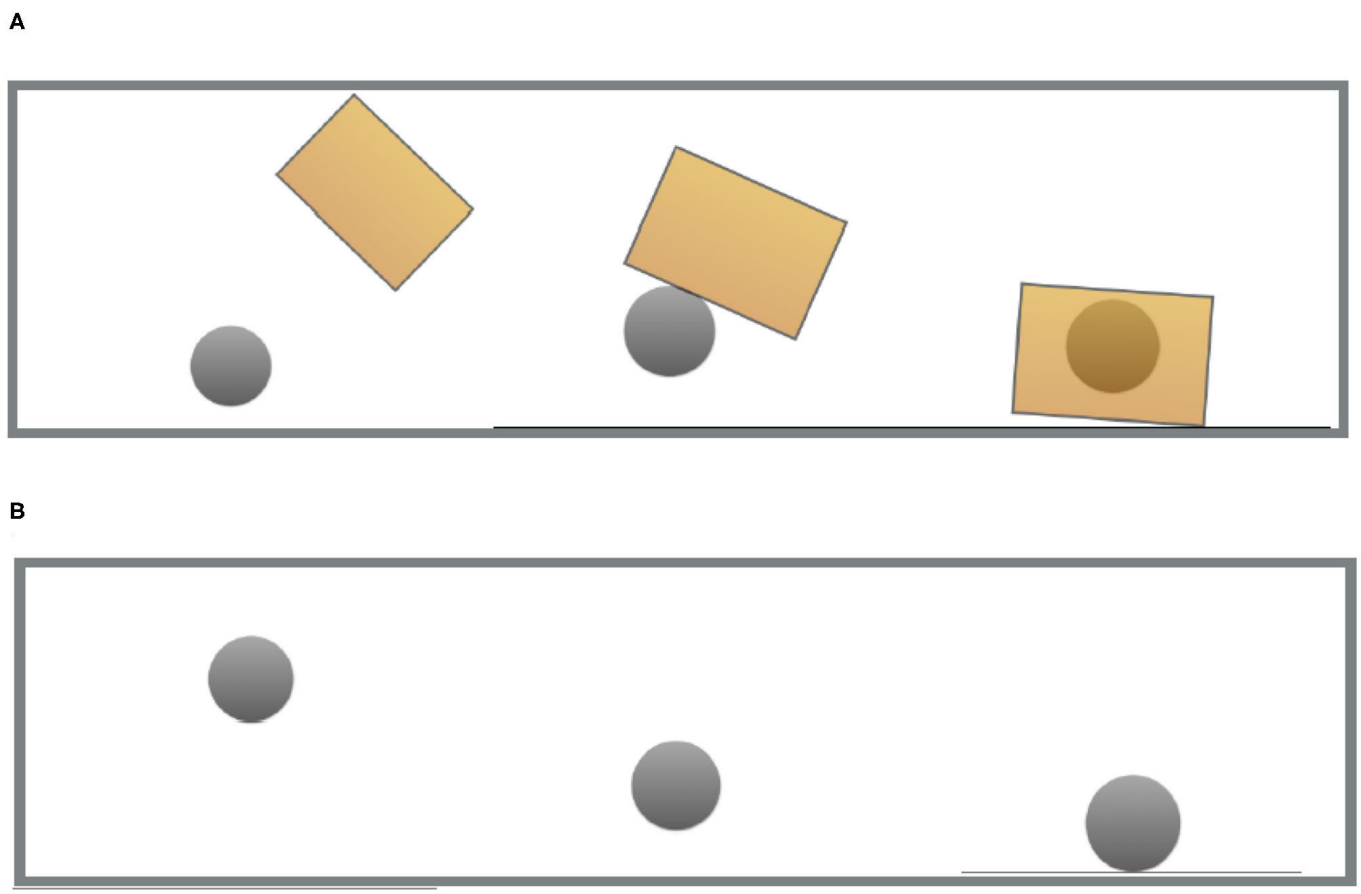
Stills from the video used in the “match” and “mismatch” conditions of experiments 1–4. **(A)** Visual prime—Containment. **(B)** Visual prime—Non-containment.

Two critical videos were created by animating individually created images with proprietary video editing software. Each video showed a ball bouncing with identical motion: In one of them (used in the “match” conditions) the ball was seen to be captured by a moving box, forcing the ball to a still stand. In the other (used in the “mismatch” conditions), the ball bounces freely and stops on its own. Figure 1 shows a series of stills for each of the videos. The videos themselves can be seen in full length in the Supplementary Material.

Furthermore, inspired by Experiment 1 of Guerra and Knoeferle (2014), two versions of each video were created: One with a printed word from each critical sentence on the ball and one without any printed word. Participants thus saw, for example, a video of a box trapping a ball (or a ball bouncing freely) that had the word *opinion* written on it, and subsequently read sentence (3), in which an “opinion” is said to be *fenced in*. This was done to maximize the possibility that participants would establish a relation between the visual context and the written sentence.

For the filler trials, four other animated videos were created that were randomly paired with the 72 filler sentences. To prevent participants from identifying the critical videos, the filler videos presented the same objects as the critical ones, i.e., a combination of bouncing balls and boxes. In the filler videos a box lands next to a bouncing ball without trapping it (filler video 1); two balls cross each other diagonally and bounce toward each other (filler video 2) or away from each other (filler video 3); and two balls fall on top of a box but only one of the balls goes in the box (filler video 4).

#### 3.2.5. Comprehension Questions

To investigate the role of literal features after a metaphor has been comprehended, we included a comprehension question after every trial. For critical trials, the question was always about the video, either (a) referring to the ball (*Was the ball in the box?*) or (b) to the metaphoric topic that may or may not have appeared written on the ball in the video (*Was the opinion in the box?*). Trials with incorrect answers were discarded from the analysis.

The idea of having these two different questions was that they might allow us to investigate different ways in which literal features could be activated after metaphor comprehension: It could be the case that literal features are simply activated because they are seen in the video and mentioned in the sentence, in which case question (a) should be easier to respond to when the video-prime seen prior to the metaphor activates the literal feature of containment. This would be compatible with indirect comparison views and with Carston's (2010) “lingering” of the literal meaning view. Alternatively, literal features could remain activated because they are part of a network of systematic mappings between topic and vehicle established during structural alignment, as suggested by Gentner and Boronat (1992), Gentner et al. (2001), and Thibodeau and Durgin (2008). This would result in a facilitation effect when answering question (b), considering that it suggests a parallel in structure between video and sentence by effectively “blending” together both representations. Finally, it could be the case that literal features are always suppressed after metaphor comprehension, in which case neither type of question should be easier to answer when the video activates the literal feature of containment compared to when the video does not activate it. This would be compatible with the category inclusion view (Glucksberg, 2008). We return to these positions and how they relate to the experimental design when discussing the results of the question-response times.

### 3.3. Design

Experiment 1 had a 2 × 2 × 2 Latin square design with three factors: “containment” (match vs. mismatch), “question type” (video-question vs. noun-question), and “prime type” (animation-prime vs. mixed-prime). “Containment” refers to whether the video showed the ball bouncing freely (mismatch conditions) or being trapped by a box (match conditions) (see Figure 1). “Question type” refers to whether the comprehension question inquired about the video (video-question conditions) or about the metaphoric topic [*the opinion* in (3)] (noun-question conditions). Finally, “prime type” refers to whether the metaphoric topic was written on the ball (mixed-prime conditions) in the video prime or whether the video prime had no written language in it (animation-prime conditions).

We calculated three eye-tracking measures commonly associated with different temporal processing stages (see Rayner, 1998, 2009) for our three regions of interest (i.e., the adjective, the noun, and the verb region): First-pass reading times, defined as the duration of all fixations made in a region until the first time the region is abandoned either to a subsequent or to a prior word; regression path duration, defined as the duration of all fixations from the first fixation in a region up to (but excluding) the first fixation to the right of this region (but including the duration of all fixations made to the left of the critical region after the first fixation in the critical region); and total reading times, defined as the sum of the duration of all fixations in a critical region. These three measures were chosen since they can provide insight about the point in time in which effects might arise: If effects are found in first-pass reading times, it would suggest that they occur during the earliest stages of processing. If they are visible in regression path duration, it would likely point to it being related to the way in which a region is integrated into the sentential context, whereas if they are found only in total reading times, it would suggest that such an effect might appear incrementally but only during later processing.

### 3.4. Predictions by Region

Our first set of predictions concerns the effect of the video on reading comprehension. We focused on three specific regions which we believed to be likely to interact with the visual prime: the adjective, noun, and verb regions.

#### 3.4.1. Adjective Region

In Guerra and Knoeferle (2014) the authors found that visually depicted spatial distance facilitated reading comprehension of adjectives denoting abstract similarity. They reasoned that this facilitation effect might be due to an existing co-indexing link between spatial distance (close, far) and semantic distance (similar, dissimilar). They borrowed this idea from Conceptual Metaphor Theory, which hypothesizes the existence of such a link (Lakoff and Johnson, 2008). This theory also posits the existence of a link between the concepts of difficulty and containment. Thus, watching videos of spatial containment might ease processing of an adjective denoting difficulty. We therefore reasoned that if there is a link between difficulty and containment similarly to that found for the case of similarity and distance, we should find a main effect of containment in the adjective region, with shorter reading times in the match vs. mismatch conditions.

#### 3.4.2. Noun Region

By adding the word in the noun region to the video (mixed prime type conditions), we expected a clear repetition priming effect to appear when participants encountered this word in the sentence. Concretely, if participants were able to integrate the written word from the video with the subsequently read sentence, we should observe a main effect of prime type in all dependent measures, with the mixed-prime conditions being overall faster to read than the animation-prime conditions.

#### 3.4.3. Verb Region

Our predictions for this region are derived from the debate on metaphor processing presented in section 2. We expected a facilitation effect on an early measure, such as first-pass reading times, provided that the video relates to the literal meaning of the verb. This finding would suggest that features related to the literal meaning of a verb (in this case, physical containment) are initially active even though they might be absent from the intended metaphoric meaning. This would be in line with the results of Weiland et al. (2014), who observed that masked primes made up of irrelevant features of the metaphoric vehicle reduced the N400 effect found upon encountering the metaphoric vehicle, and would also generally support the indirect comparison view of metaphor understanding.

Alternatively, if activating the spatial representation of containment interferes with processing the metaphorically used verb, we should find longer reading times in the match vs. mismatch conditions. This would be more in line with the findings of McGlone and Manfredi (2001) and generally with category inclusion accounts that claim that literal features irrelevant for understanding the metaphor are actively suppressed during processing. Activating them should therefore interfere with the construction of metaphoric meaning.

### 3.5. Post-sentence Comprehension Question

A second set of predictions relates to how understanding each metaphor affects participants' response time patterns for questions related to the content of the video.

The main prediction for the response patterns to the post-comprehension questions was that if the feature of physical containment is active after participants have understood the sentence, it should be possible to find a main effect of containment on question-answering times, with overall shorter answering times in the match vs. mismatch conditions. This would suggest that the feature of containment activated in the match conditions (the ball is trapped by the box) was not suppressed after the metaphor was understood and facilitates answering both question (a) *Was the ball in the box?* and (b) *Was the opinion in the box?* If, on the other hand, the features activated by the video are suppressed after the metaphor has been understood, there should be either an interference or a null-effect of containment on response times.

However, given that there were two types of post-sentence comprehension questions, (a) and (b) above, it would be possible to observe different result patterns beyond the prediction of a main effect of Containment. Such patterns would bring about a more nuanced view on the activation of literal features after a metaphor has been comprehended, which could further inform theories of metaphor comprehension. Table 1 presents a description of all conditions for the response times.

**Table 1:** Description of all conditions for the question-response times in Experiments 1, 2, and 3.

**Condition**	**Question asked**	**Description of video-prime**	**Correct response**
Noun-question, animation-prime, match	Was the opinion in the box?	Ball is trapped in a box	No
Video-question, animation-prime, match	Was the ball in the box?	Ball is trapped in a box	Yes
Noun-question, mixed-prime, match	Was the opinion in the box?	Ball with word “opinion” on it is trapped in a box	Yes
Video-question, mixed-prime, match	Was the ball in the box?	Ball with word “opinion” on it is trapped in a box	Yes
Noun-question, animation-prime, mismatch	Was the opinion in the box?	Ball is bouncing freely	No
Video-question, animation-prime, mismatch	Was the ball in the box?	Ball is bouncing freely	No
Noun-question, mixed-prime, mismatch	Was the opinion in the box?	Ball with word “opinion” on it is bouncing freely	No
Video-question, mixed-prime, mismatch	Was the ball in the box?	Ball with word “opinion” on it is bouncing freely	No

Of particular importance for a nuanced view on the role of literal features are the response times in the noun-question/animation-prime conditions. This is because, in these conditions, participants were asked a question that effectively “blended” the representations of video and sentence by asking whether the “opinion” was in the box when there was nothing written on the ball in the video but they had read about an opinion in the sentence.

If the feature of physical containment is activated after sentence comprehension, we would expect this feature to interfere with correctly answering the question in the noun-question/animation-prime conditions (because the correct response here would be NO and participants might want to answer YES if the feature of Containment is active), particularly in the match condition, where physical containment was seen in the video. This should in turn result in an interaction of question type and prime type, with the noun-question/animation-prime conditions showing longer reaction times than all other conditions. If the match level (of the noun-question/animation-prime conditions) is harder to respond to than the mismatch level, there should additionally be a three-way interaction between question type, prime type, and containment. If, on the other hand, the feature of physical containment is not active after participants have understood the sentence, we should expect the noun-question/animation-prime conditions to take just as long as the others, thus not resulting in a significant interaction of question type and prime type.

### 3.6. Procedure

**Figure 2:**
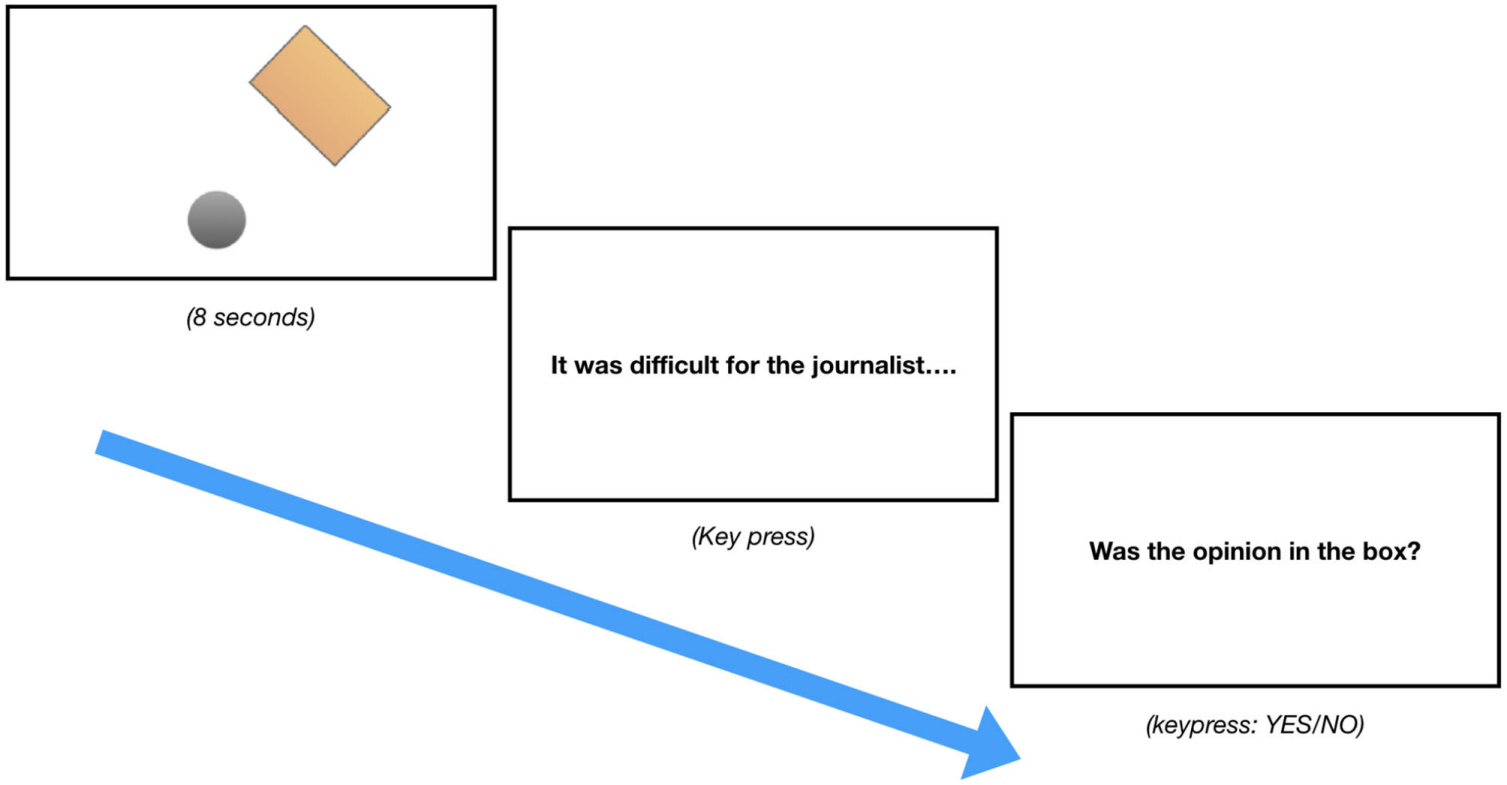
Example of the progression of a trial in experiments 1–3.

Participants' eye movements were recorded using an Eyelink 1000 plus desktop head-stabilized tracker, produced by SR Research. At the beginning of each experimental session, the eye-tracker was calibrated with a 9-point calibration procedure to ensure accurate monitoring of the eyes. The procedure was performed and repeated until there was less than a maximum error of 0.5°. If it was not possible to meet this criterion, the experiment was aborted and participants were replaced. Re-calibration was performed after every block, i.e., twice more. After calibration, participants saw three practice trials before the experiment began. Each trial in the experiment consisted of three phases (see Figure 2): First, participants saw an animated video presented on the screen for 8 s. The video disappeared and a sentence appeared on the screen. Participants read the sentence and pressed a button on a Cedrus response pad that was in front of them when they had finished reading. The sentence then disappeared and a question appeared on the screen. Participants had to answer this question by pressing either the YES or NO button on the pad (position of YES and NO buttons was counterbalanced across participants). An entire experimental session lasted an average of around 50 min.

### 3.7. Analysis and Results

#### 3.7.1. Analysis of Eye-Tracking Data

Prior to analysis, an intercepts-only regression model was fitted to the data in order to observe the distribution of the residuals. These were not normally distributed (which violates the assumptions of the linear model), and thus a box-cox test (Box and Cox, 1964) was performed. The test showed that the reading times measures needed to be transformed using a Lambda value of −0.7, which was used for transforming all eye-tracking measures and regions. Cases in which participants gave an incorrect answer to the comprehension question were also excluded from all analyses. This procedure was followed for all subsequent experiments. Accuracy for comprehension questions in experiment 1 was above 85% in all conditions.

We analyzed all data in our experiments using the R statistical programming environment and the LME4 package for regression analysis. To test our predictions, we fitted mixed-effects linear regression models to every measure and every region. For constructing the statistical models, we followed the recommendations of Barr et al. (2013). First, we tried fitting the largest possible random effects structure granted by our experimental design (in our case, random intercepts and slopes by items and subjects for both independent variables). If the model failed to converge, we reduced the random effects structure step-wise until a converging model was found by first removing the random correlations, then the random intercepts, followed by the interaction effects and the main effects. We used the same maximally converging random effects structure for all dependent measures in every region.

All models included trial order as a fixed effect, since it significantly improved the model fit. The models were fitted using a sum-contrast coding scheme (unless stated otherwise). Alpha thresholds for assessing statistical significance for eye-tracking data were Bonferroni-corrected, following the recommendations of von der Malsburg and Angele (2017).

**Figure 3:**
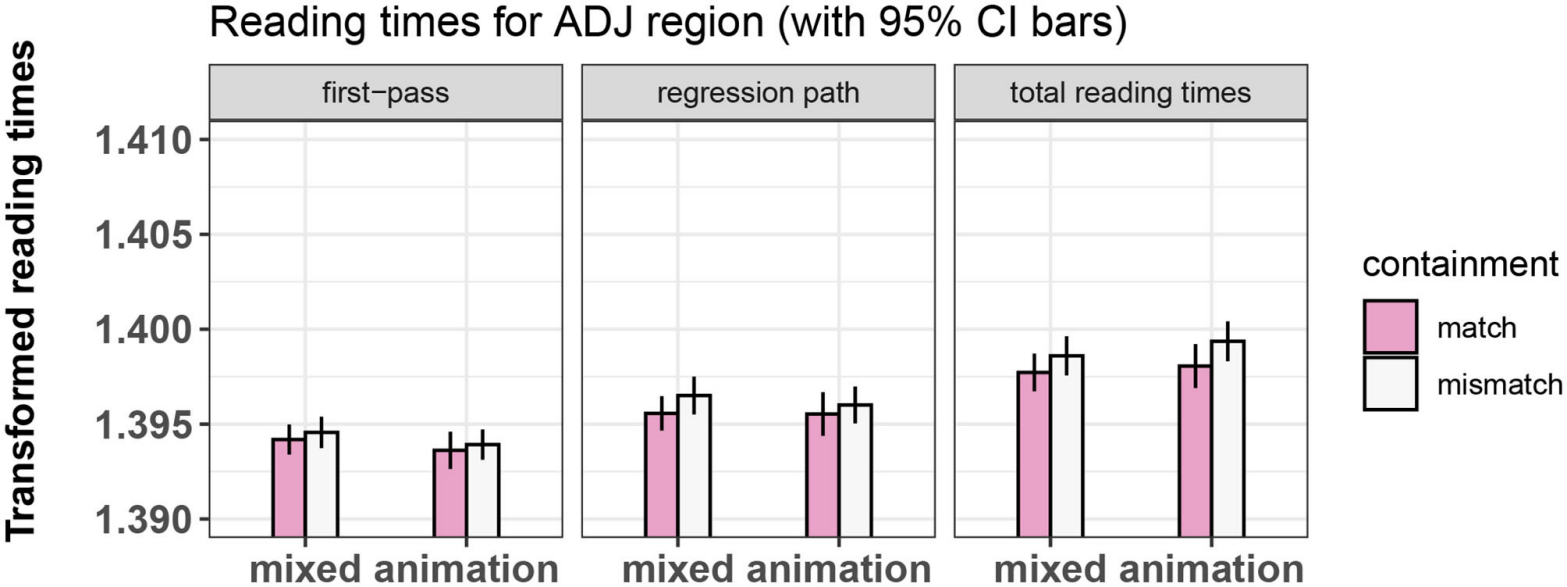
Summary of results for the ADJ region, Experiment 1.

**Figure 4:**
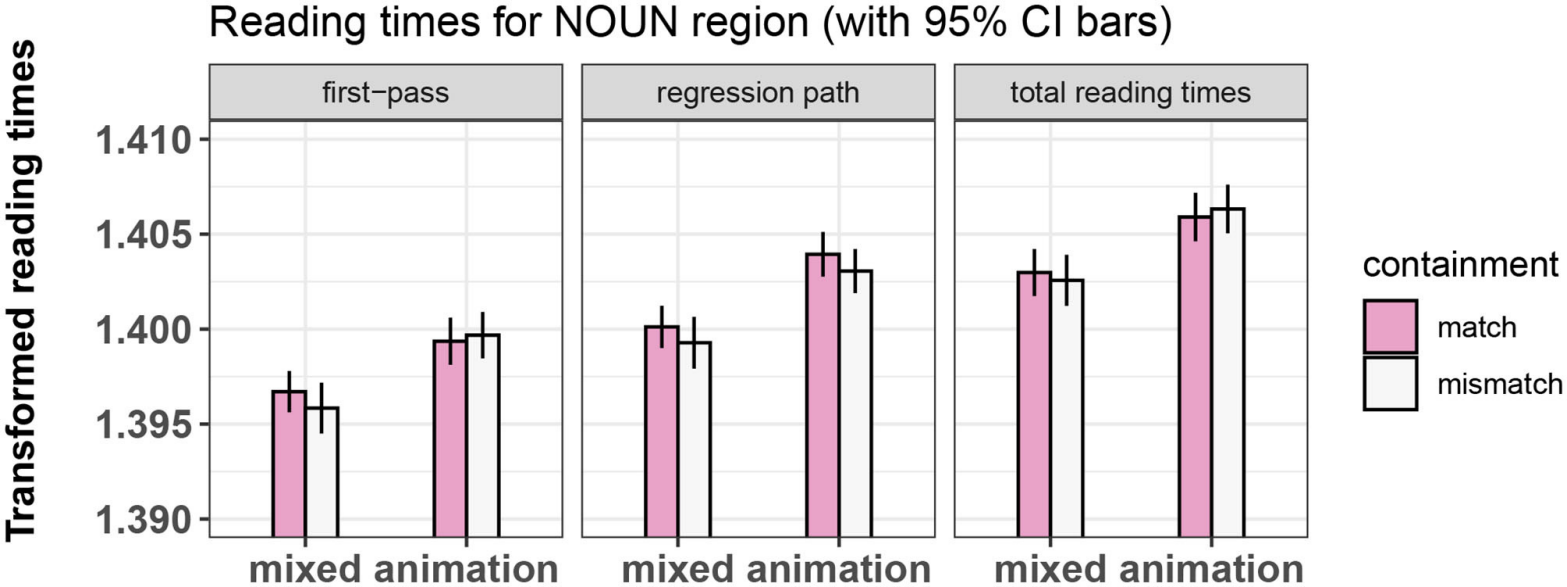
Summary of results for the NOUN region, Experiment 1.

**Figure 5:**
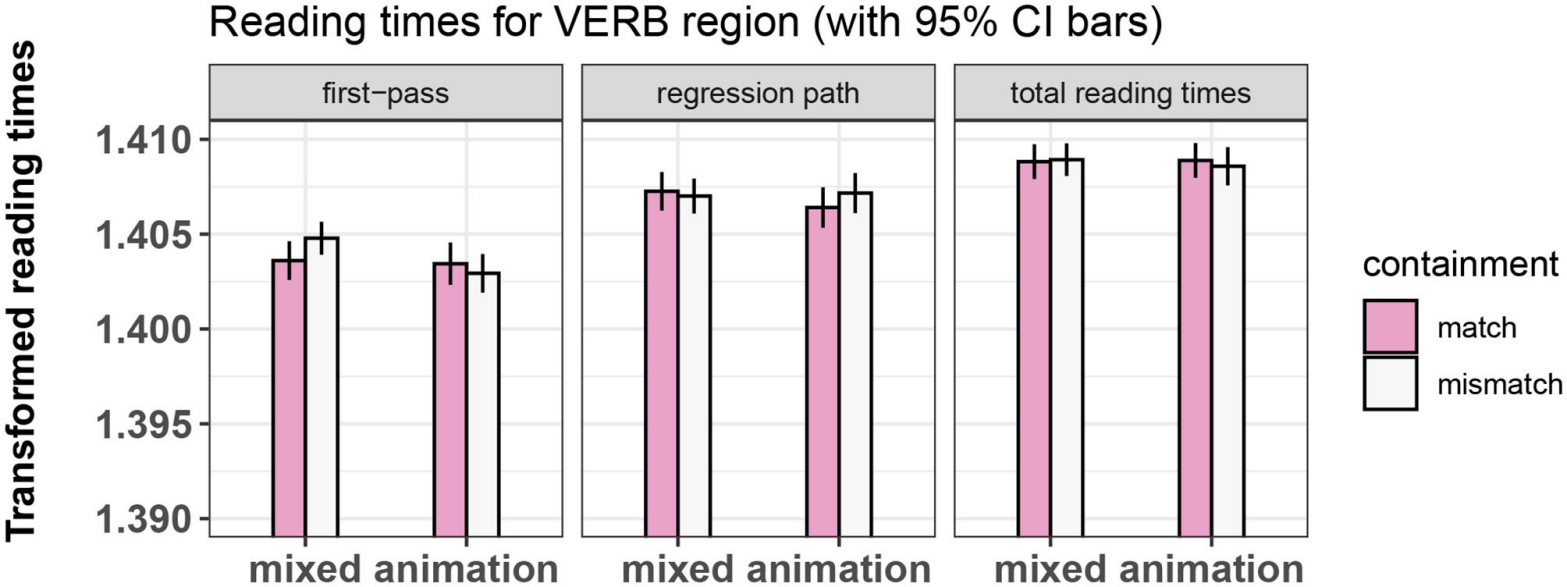
Summary of results for the VERB region, Experiment 1.

**Figure 6:**
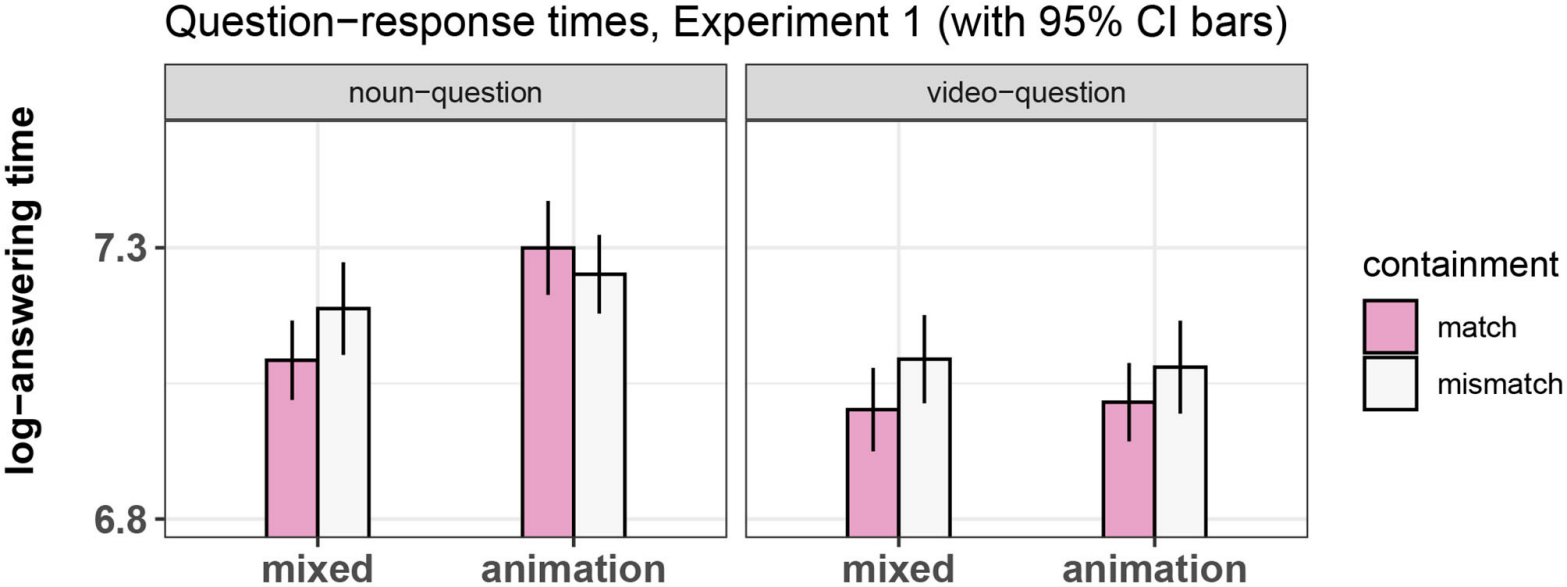
Summary of results for the question response time, Experiment 1.

The final random effects structure used for every model is shown in Table 2. Figures 3–5 show bar-plots of the results in the adjective, noun, and verb region respectively. The output of the respective statistical models can be seen in Tables 3–5. Figure 6 shows the results of the post-sentence comprehension question response times.

**Table 2:** Random effects structure for models in every experiment.

	**Models of eye-tracking data**	**Models of forced choice data**
Experiment_1	(0 + prime type * containment ||item) + (0 + prime type * containment || subject)	(1 + type || subject) + (0 + type | item)
Experiment_2	(0 + prime type * containment || item)+ (0 + prime type * containment || subject)	(1 + type || subject) + (0 + type | item)
Experiment_3		(0 + question type * prime type * containment || item) + (0 + containment * question type * prime type ||subject)
Experiment_4		(1|subject) + (1|item)

**Table 3:** Regression analysis of reading times in the ADJECTIVE region of Experiment 1.

	**Dependent variable**		
	**First-pass**	**Regression path**	**Total reading times**
Prime Type	0.0003	0.0001	−0.0002
	t = 1.444	t = 0.581	t = −0.795
Containment	−0.0002	−0.0003	−0.0005
	t = −0.744	t = −1.389	t = −1.956
Trial order	−0.00000	−0.00001	−0.00003
	t = −0.293	t = −1.512	t = −3.105^**^
Containment/Prime Type interaction	−0.00002	−0.0001	0.0001
	t = −0.107	t = −0.494	t = 0.338
Constant	1.394	1.397	1.400
	t = 3,233.003^***^	t = 2,739.009^***^	t = 2,625.450^***^
Observations	1,180	1,180	1,180
Log Likelihood	4,162.329	3,965.666	3,910.930
Akaike Inf. Crit.	−8,300.659	−7,907.333	−7,797.859
Bayesian Inf. Crit.	−8,239.780	−7,846.453	−7,736.980

^*^p < 0.017;

** p < 0.0033;

*** *p < 0.00033*.

*Significance thresholds are Bonferroni-corrected (alpha/3)*.

**Table 4:** Regression analysis of reading times in the NOUN region of Experiment 1.

	**Dependent variable**		
	**First-pass**	**Regression path**	**Total reading times**
	**(1)**	**(2)**	**(3)**
Prime Type	−0.002	−0.002	−0.002
	t = −5.490^***^	t = −6.144^***^	t = −5.230^***^
Containment	0.0002	0.0004	0.00003
	t = 0.430	t = 1.221	t = 0.075
Trial order	−0.00000	−0.00001	−0.00004
	t = −0.072	t = −1.265	t = −3.687^***^
Containment/Prime Type interaction	0.0003	−0.00002	0.0002
	t = 0.800	t = −0.054	t = 0.507
Constant	1.398	1.402	1.407
	t = 2,294.932^***^	t = 2,344.835^***^	t = 2,192.578^***^
Observations	1,111	1,111	1,111
Log Likelihood	3,559.108	3,578.840	3,505.177
Akaike Inf. Crit.	−7,094.215	−7,133.679	−6,986.355
Bayesian Inf. Crit.	−7,034.059	−7,073.523	−6,926.198

^*^p < 0.017; ^**^p < 0.0033;

*** p < 0.00033.

*Significance thresholds are Bonferroni-corrected (alpha/3)*.

**Table 5:** Regression analysis of reading times in the VERB region of Experiment 1.

	**Dependent variable**		
	**First-pass**	**Regression path**	**Total reading times**
	**(1)**	**(2)**	**(3)**
Prime Type	0.001	0.0002	0.0002
	t = 2.180	t = 0.760	t = 0.792
Containment	−0.0001	−0.0001	0.0001
	t = −0.496	t = −0.459	t = 0.611
Trial order	−0.00002	−0.00001	−0.0001
	t = −2.234	t = −1.383	t = −9.256^***^
Containment/Prime Type interaction	−0.0004	0.0002	−0.0002
	t = −1.683	t = 0.891	t = −0.736
Constant	1.405	1.408	1.413
	t = 2,726.656^***^	t = 2,704.316^***^	t = 3,084.624^***^
Observations	1,148	1,148	1,148
Log Likelihood	3,877.586	3,866.024	4,012.204
Akaike Inf. Crit.	−7,731.172	−7,708.049	−8,000.409
Bayesian Inf. Crit.	−7,670.623	−7,647.499	−7,939.859

^*^p < 0.017; ^**^p < 0.0033;

*** *p < 0.00033*.

*Significance thresholds are Bonferroni-corrected (alpha/3)*.

#### 3.7.2. Results of Eye-Tracking, Adjective Region

No significant main effects or interactions were found in any measure for this region.

#### 3.7.3. Results of Eye-Tracking, Noun Region

As predicted, we observed a significant main effect of prime-type in all three measures, with shorter reading times in the mixed-prime vs. animation-prime conditions. This confirms that our experimental paradigm was sensitive enough to detect identity priming effects, and that participants were actively integrating the information processed during the video with the information from the sentence.

#### 3.7.4. Results of Eye-Tracking, Verb Region

No significant main effects or interactions of our manipulated variables were found in any measure for this region.

#### 3.7.5. Analysis and Results of Question Response Times

A box-cox test determined that the response times needed to be log-transformed. We thus fitted a linear mixed-effects regression model to the log-transformed reaction times. This model was fitted only to correct responses, which were over 92% of all trials. The results pattern can be seen in Figure 6 and the output of the model is summarized in Table 6.

**Table 6:** Regression analysis of response-times in Experiment 1.

	**Dependent variable**
	**Response times (in log-milliseconds)**
Containment	−0.021
	t = −1.813
Prime Type	−0.027
	t = −2.325^*^
Question Type	0.081
	t = 6.754^***^
Trial order	−0.004
	t = −9.385^***^
Containment/Prime Type interaction	−0.027
	t = −2.267^*^
Containment/Question Type interaction	0.012
	t = 1.032
Question Type/Prime Type interaction	−0.038
	t = −3.269^**^
Three-way interaction	−0.016
	t = −1.345
Constant	7.327
	t = 215.365^***^
Observations	1,111
Log Likelihood	−559.837
Akaike Inf. Crit.	1,145.674
Bayesian Inf. Crit.	1,210.843

* p < 0.05;

** p < 0.01;

*** *p < 0.001*.

There was a main effect of question type, showing that participants were significantly slower at answering questions in the noun vs. video-question conditions. There was also a main effect of prime type, indicating that participants were faster to answer questions in the mixed-prime compared to the animation-prime condition, and a main effect of containment, showing that there was an overall facilitation in the match vs. mismatch conditions. There were also significant interactions between question type and prime type and containment and prime type, reflecting in particular that the noun-question/animation-prime conditions displayed a different pattern than all others (see Figure 6). The three-way interaction was not significant.

A potential response bias was discovered after running the experiment: The correct answer to the question asked was always NO in the mismatch conditions and YES in the match conditions (see Table 1). It is therefore not possible to tell whether the effect of containment was caused by the difference in the conditions (match vs. mismatch) or by the differences in correct answer (YES vs. NO).

The noun-question/animation-prime was the only exception to this: Here, the correct response was NO in both match and mismatch levels. Because of this, we re-fitted the statistical model for the question-response times using a treatment contrast coding scheme in order to look at the noun-question/animation-prime condition exclusively. This was important because both match and mismatch levels of this condition were the only ones where both the question (“Was the NOUN in the box?”) and the correct answer (NO) were the same. This type of contrast coding allows for direct comparisons between the condition set as the intercept of the model and the other individual conditions. This model showed no significant difference between match and mismatch levels of the noun-question/animation-prime. This model is shown in **Table 12**.

### 3.8. Discussion

In Experiment 1, we failed to find a difference in reading times between conditions in the adjective region. More importantly, we found no differences in the verb region, the main interest region of the experiment. However, the presence of the effect of priming type in the NOUN region suggests that the absence of an effect of containment might be interpreted meaningfully: It could be the case that we did not find an effect of containment on reading times of the verb because the feature of containment is not relevant for the construction of the metaphoric meaning and it is thus ignored during processing, exerting neither facilitation nor interference. This interpretation would be broadly compatible with views that ascribe an insignificant role to features related exclusively to the encoded meaning of the metaphoric vehicle during processing.

However, it might also be possible that no effect was found given the temporal distance between presentation of the visual prime and reading of the metaphorically used verb. Perhaps this distance masked a true facilitation or interference effect that the video would have otherwise exerted on processing the verbs. This lays the groundwork for Experiment 2, in which we changed the sentence structure so that the verb could be temporally closer to the video prime.

Results from the post-sentence comprehension questions present an intricate pattern. The results showed a main effect of question type, with longer response times in the noun-question conditions than in the video-question conditions. There was a main effect of containment, with shorter response times in the match compared to the mismatch conditions in all but the noun-question/animation-prime conditions (as evidenced by the interaction effect between containment and question type).

To better understand this pattern, it is useful to think about how the results might possibly be linked to the theoretical debate on the activation of literal features following metaphor comprehension. Indirect comparison views suggest that after a metaphor is understood, literal features remain active because they are part of the network of established mappings between topic [in this case, the target noun *opinion* in sentence (3)] and vehicle [the verb *fenced in* in (3)], which can be used to reason analogically about subsequent linguistic input (see for example Gentner et al., 2001). If this holds, it would accommodate a facilitation effect of match vs. mismatch levels in the video-question conditions, signifying a sustained activation of the feature “containment.” It would also account for an interference effect of match vs. mismatch levels in the noun-question/animation-prime conditions, which could be explained as a sustained activation of established mappings between different conceptual domains which interferes with answering a question about an “opinion” being in the video. This is because question (a) is a reference to the video alone, requiring only information about the feature of containment in order to answer it. If the feature is active, this should result in a facilitation effect compared to when containment was not presented (i.e., the mismatch condition). Question (b), on the other hand, is a complex combination of information about the sentence (given the presence of the target noun) and the video (given the reference to the box, which could have only been seen in the video). In this case, an interference effect for answering question (b) in the match vs. mismatch conditions would suggest that not only the feature of containment has been activated (as would be the case in the video-question conditions), but also its relationship with the metaphoric topic (the target noun). This should cause difficulty when negatively answering a question about an “opinion” being in the box. Carston (2010) suggests that literal features might “linger” after a metaphor has been understood. However, her theory seems to suggest that they “linger” only as semantic features, not as part of a network of systematic associations between topic and vehicle. That being the case, it would explain a facilitation effect of match vs. mismatch video on the question-response times in the video-question/animation-prime condition, but there should not be an effect on the response times in the noun-question/animation-prime conditions.

At first glance then, the pattern of results found in Experiment 1 seems to be in line with the idea that when the conceptual feature of containment was activated by the verb, it generally facilitated responses, resulting in shorter response times in the contained vs. not-contained conditions in all but the noun-question/animation-prime conditions.

This could suggest that the feature of containment was activated after the metaphor was understood, but not as part of a complex mapping between containment and the metaphoric topic (which would have caused a difference in the noun-question/animation-prime conditions), compatible with Carston's (2010) view on the “lingering” of the literal meaning, but incompatible with the stronger view of Gentner et al. (2001), according to which the pattern of mappings should remain available for further processing and potentially cause interference with the answering of the question.

There is, however, a simpler explanation for the current pattern of results. As mentioned in the results section, the correct responses were confounded with the match and mismatch conditions, with match conditions always requiring a YES response and mismatch conditions a NO response in all but the noun-question/no-label conditions, were the correct response was NO in both levels of containment. It is therefore likely that it was simply easier for participants to answer YES than to answer NO, explaining the main effect of containment. Additionally, the effect of question type could be due to the fact that questions in the “noun” conditions (which varied according to the target noun in every trial, 33 characters on average) were on average longer than the questions in the “video” conditions (which were always the same, i.e., *Was the ball in the box?*, 30 characters in German). It is possible that participants just took longer to read the questions in the noun compared to the video conditions and thus took longer to answer the question.

The only comparison not affected by these two issues was that between match and mismatch levels of the noun-question/animation-prime condition. For these two levels, the question and correct response remained the same. We found no significant difference between these two conditions. It's important to note, however, that the YES/NO confound affected only the question response times and not the eye-tracking data. We address the issue of the interpretation of question-response times in Experiment 3, where we examine the response patterns to the same questions in the absence of metaphorical verbs. For now, we turn to Experiment 2, where we attempted to replicate the pattern of reading times displayed in Figure 5 using sentences with a different syntactic structure.

## 4. Experiment 2

The goal of Experiment 2 was to determine the robustness of the results of Experiment 1. First, we altered the sentence structure in order to minimize the temporal distance between prime and verb. We did this because we thought it was likely that participants were not able to use the information extracted from the visual prime to facilitate processing of the metaphoric verb due to working memory constraints. This possibility finds some support in the literature on working memory, where it has been noted that people have a relatively low average number of sequentially presented meaningful units that they can remember (somewhere between 3 and 7, Miller, 1956; Chen and Cowan, 2005). We also increased the number of participants, from 48 to 64, to obtain higher statistical power. We did this following a power analysis via simulation using the R package SimR (Green and MacLeod, 2016). For the power analysis, we took the model of the total reading times for the verb region as starting point. The simulations suggested that with 64 participants we would have over 80% power to detect a main effect of containment, assuming a true effect size of containment of Cohen's *d* = 0.15, i.e., somewhat smaller than the rule of thumb for a “small” effect size (Sawilowsky, 2009). By doing this we aimed to either detect a small effect that we were not able to find in the previous experiment, or to replicate the pattern of results of Experiment 1 with more validity.

### 4.1. Participants

Sixty-four native speakers of German (ages 18–31, 39 female) with normal or corrected-to-normal vision were recruited and tested at the Humboldt-Universität zu Berlin. None of them had participated in Experiment 1. They gave their informed consent and received 8 euros as compensation upon finishing the experiment. Experiment 2 was covered by the ethics vote granted to the psycholinguistics lab of the Humboldt-Universität zu Berlin by the German Linguistic Society (Deutsche Gesellschaft für Sprachwissenschaft, DGfS).

### 4.2. Materials, Design, and Procedure

The materials, design, and procedure were identical to those in Experiment 1, except for the syntactic structure of the critical sentence, which now displayed a leftward movement of the subject clause. This allowed for the verb to appear as the fourth word in the sentence, making it temporally closer to the video prime. The structure of the sentences was as follows:

(4) *Dass seine / Meinung* *target noun* */ umgittert* *verb**/ wurde nach dem Regimewechsel, war / schwierig* *adj* */ für den Redakteur*.
  “*That his / opinion* *target noun* */ fenced-in* *verb* */ was after the change in regime, was /difficult* *adj**/ for the journalist”*

“The fact that his opinion was fenced-in after the change in regime was difficult for the journalist.”

### 4.3. Predictions

Our predictions were motivated by the results of Experiment 1: If the absence of an effect of containment on the verb region was due to the temporal distance between verb and video, moving the verb closer to the video should correct this. Specifically, if priming physical containment facilitates processing of verbs of spatial containment used metaphorically, we should find shorter reading times in the match vs. mismatch conditions in the VERB region.

With regards to the question-answering times: The overall facilitation effect of match vs. mismatch in Experiment 2 was confounded with the type of response (“YES” for matches and “NO” for mismatches) in all but one relevant comparison: The noun-question/animation-prime conditions. We did not find a significant difference between these two conditions. In Experiment 2 we hoped to replicate the question-answering pattern in general, and the results of the noun-question/animation-prime conditions in particular.

### 4.4. Results

#### 4.4.1. Eye-Tracking

**Figure 7:**
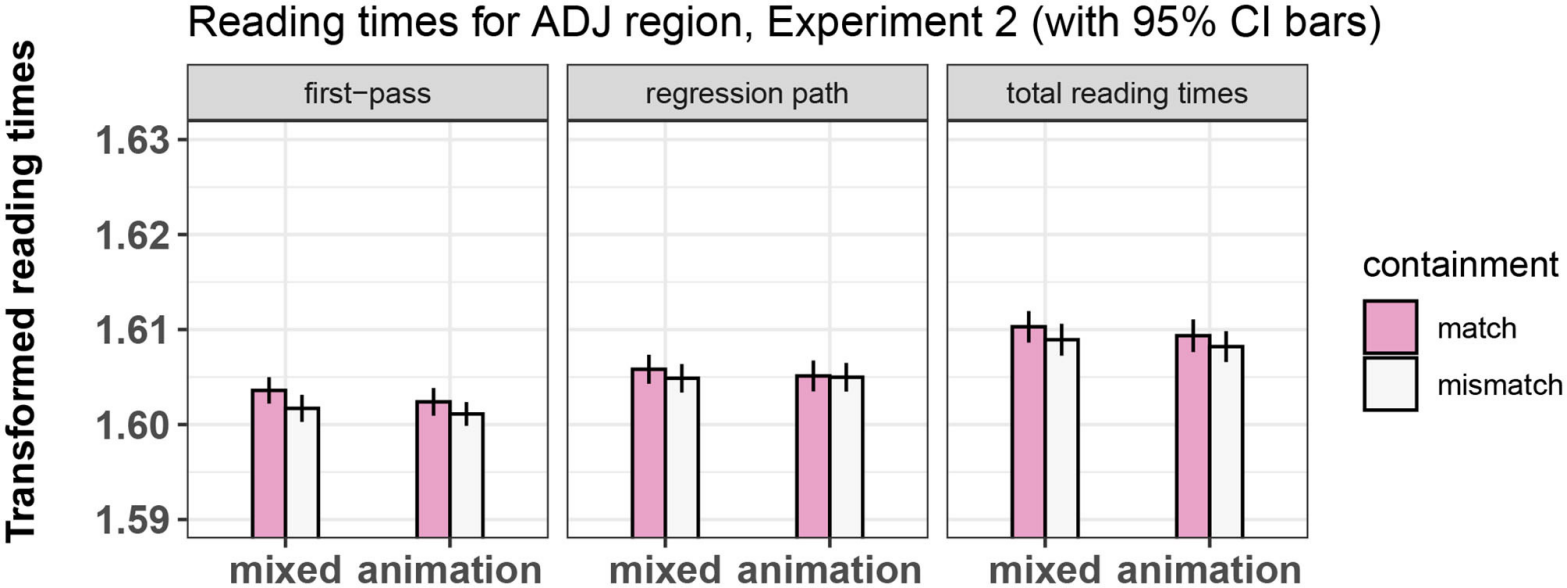
Summary of results for the ADJ region, Experiment 2.

**Figure 8:**
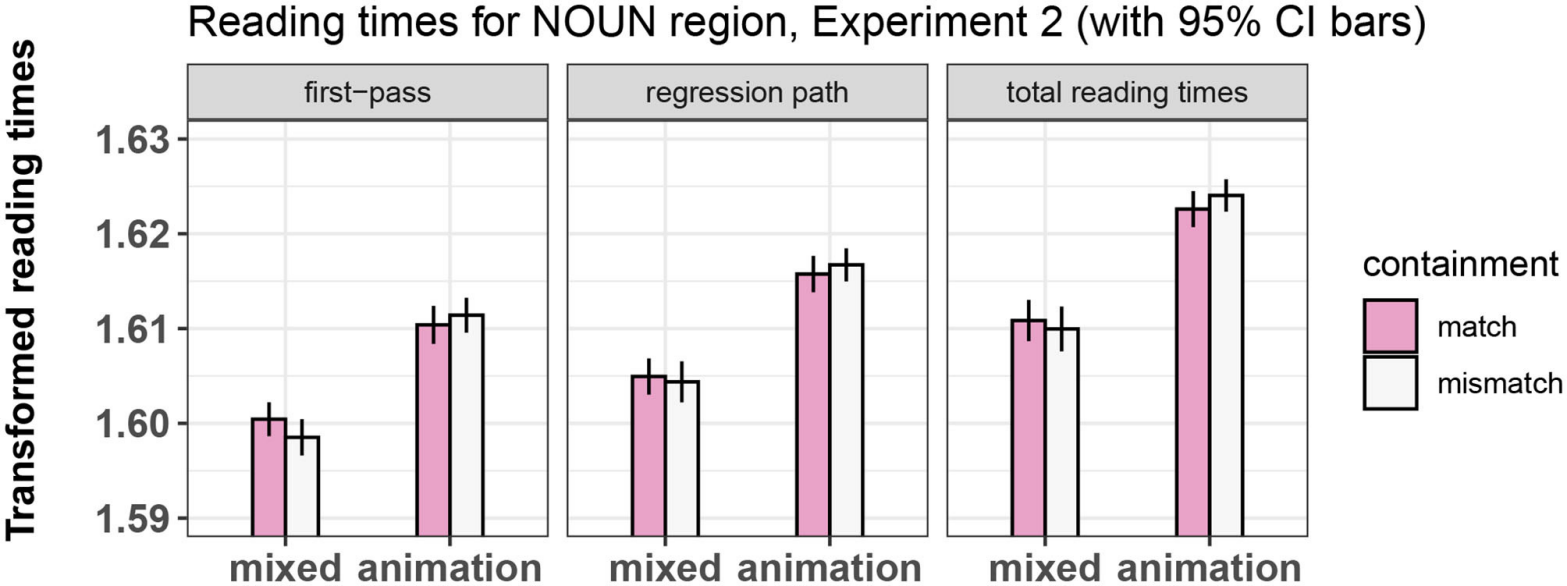
Summary of results for the NOUN region, Experiment 2.

**Figure 9:**
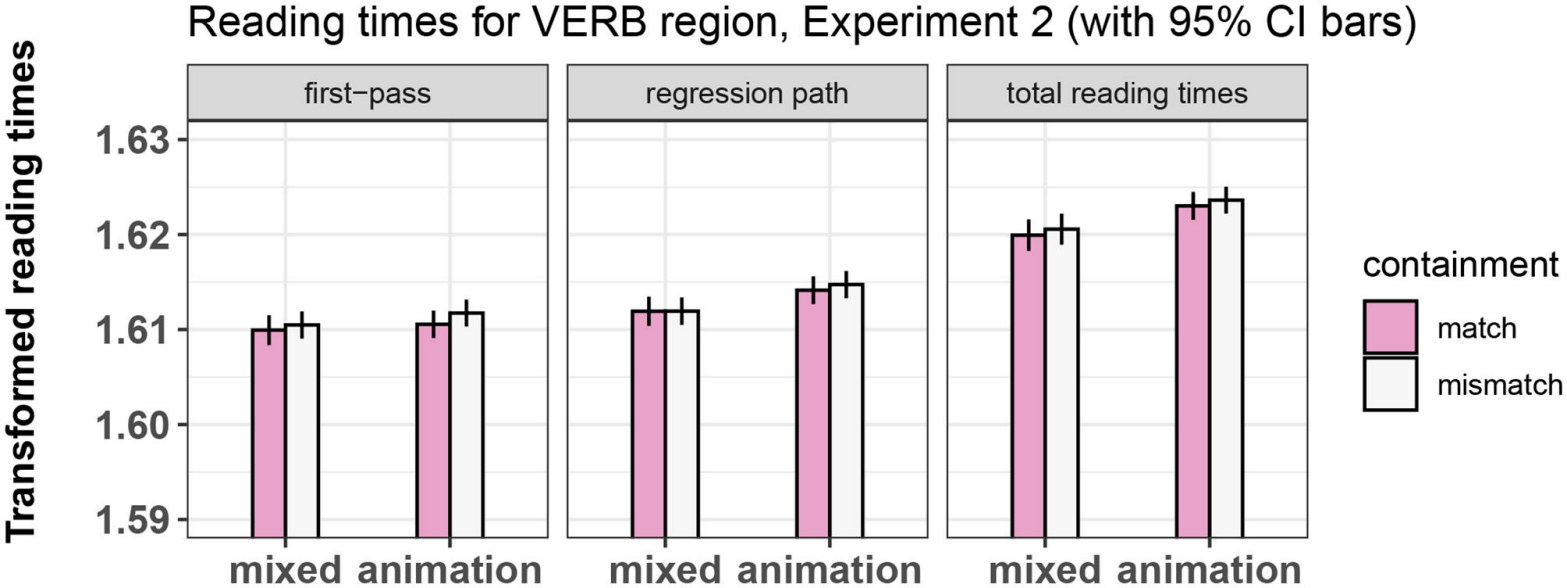
Summary of results for the VERB region, Experiment 2.

Results for all regions and measures are shown in Figures 7–9. The output of the statistical models can be seen in Tables 7–9.

**Table 7:** Regression analysis of reading times in the ADJECTIVE region of Experiment 2.

	**Dependent variable**		
	**First-pass**	**Regression path**	**Total reading times**
	**(1)**	**(2)**	**(3)**
Prime Type	0.0005	0.0002	0.0005
	t = 1.356	t = 0.445	t = 1.029
Containment	0.001	0.0002	0.001
	t = 2.329	t = 0.660	t = 1.389
Trial order	−0.00001	−0.00002	−0.0001
	t = −0.619	t = −1.480	t = −3.907^***^
Containment/Prime Type interaction	0.0001	0.0002	0.00001
	t = 0.433	t = 0.479	t = 0.026
Constant	1.603	1.606	1.612
	t = 2,303.281^***^	t = 2,069.854^***^	t = 1,926.601^***^
Observations	1,634	1,634	1,634
Log Likelihood	4,709.178	4,530.882	4,406.515
Akaike Inf. Crit.	−9,394.356	−9,037.763	−8,789.029
Bayesian Inf. Crit.	−9,329.571	−8,972.978	−8,724.244

^*^p < 0.017; ^**^p < 0.0033;

*** *p < 0.00033*.

*Significance thresholds are Bonferroni-corrected (alpha/3)*.

**Table 8:** Regression analysis of reading times in the NOUN region of Experiment 2.

	**Dependent variable**		
	**First-pass**	**Regression path**	**Total reading times**
	**(1)**	**(2)**	**(3)**
Prime Type	−0.006	−0.006	−0.006
	t = −9.825^***^	t = −10.309^***^	t = −12.163^***^
Containment	0.0002	−0.0002	−0.0002
	t = 0.465	t = −0.334	t = −0.469
Trial order	−0.00002	−0.00005	−0.0001
	t = −1.618	t = −3.415^**^	t = −4.296^***^
Containment/Prime Type interaction	0.001	0.0002	0.0004
	t = 1.021	t = 0.294	t = 0.575
Constant	1.607	1.613	1.621
	t = 1,744.954^***^	t = 1,728.147^***^	t = 1,625.652^***^
Observations	1,491	1,491	1,491
Log Likelihood	3,908.996	3,884.676	3,796.362
Akaike Inf. Crit.	−7,793.993	−7,745.352	−7,568.724
Bayesian Inf. Crit.	−7,730.306	−7,681.666	−7,505.037

^*^p < 0.017;

** p < 0.0033;

*** *p < 0.00033*.

*Significance thresholds are Bonferroni-corrected (alpha/3)*.

**Table 9:** Regression analysis of reading times in the VERB region of Experiment 2.

	**Dependent variable**		
	**First-pass**	**Regression path**	**Total reading times**
	**(1)**	**(2)**	**(3)**
Prime Type	−0.0004	−0.001	−0.001
	t = −1.165	t = −3.061^**^	t = −3.779^***^
Containment	−0.0005	−0.0002	−0.0004
	t = −1.153	t = −0.550	t = −1.129
Trial order	−0.00004	−0.00005	−0.0001
	t = −3.886^***^	t = −4.105^***^	t = −8.117^***^
Containment/Prime Type interaction	0.0001	0.0001	−0.0002
	t = 0.270	t = 0.191	t = −0.370
Constant	1.613	1.616	1.627
	t = 2,206.844^***^	t = 2,209.841^***^	t = 2,151.451^***^
Observations	1,566	1,566	1,566
Log Likelihood	4,459.908	4,456.508	4,402.584
Akaike Inf. Crit.	−8,895.816	−8,889.016	−8,781.167
Bayesian Inf. Crit.	−8,831.541	−8,824.741	−8,716.892

^*^p < 0.017;

** p < 0.0033;

*** *p < 0.00033*.

*Significance thresholds are Bonferroni-corrected (alpha/3)*.

##### 4.4.1.1. Adjective

No significant effects of containment or of prime type were found in this region.

##### 4.4.1.2. Noun

We replicated the main effect of prime type on all measures, with the mixed-prime conditions showing overall shorter reading times than the animation-prime conditions. This shows that our participants were in fact relating video to sentence, leading to a reliable priming effect.

##### 4.4.1.3. Verb

We failed to find an effect of containment on any measure, as was the case in Experiment 1. There was also no effect of prime type and no significant interaction of containment and prime type.

#### 4.4.2. Question-Response Times

**Figure 10:**
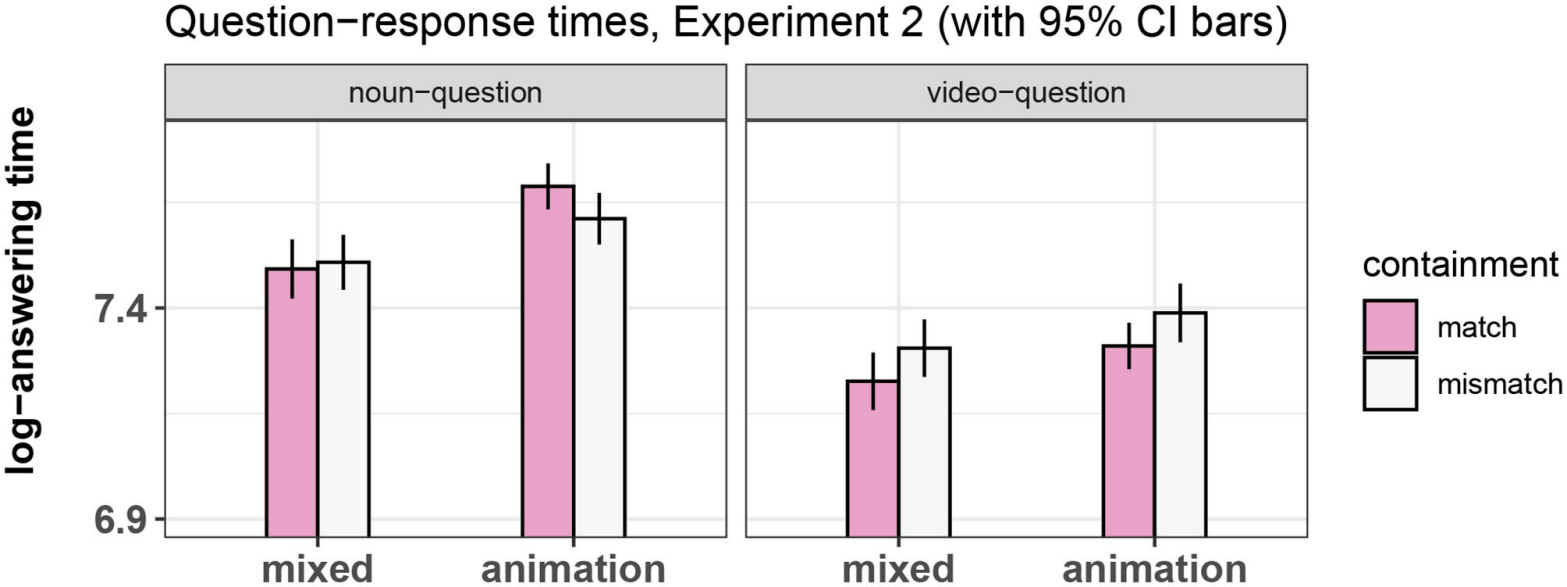
Summary of results for the question response time, Experiment 2.

Question-response times were analyzed in the same way as in Experiment 1. As can be seen in Figure 10, the results are very similar to those of Experiment 1. We replicated all previous findings with the exception of the main effect of containment: There was a main effect of question type and of prime type. There was an interaction between containment and question type and an interaction between question type and prime type. This model can be seen in Table 10.

**Table 10:** Regression analysis of response-times in Experiment 2.

	**Dependent variable**
	**Response times (in log-milliseconds)**
Containment	−0.017
	t = −1.817
Prime Type	−0.060
	t = −6.551^***^
Question Type	0.134
	t = 14.741^***^
Trial order	−0.004
	t = −13.198^***^
Containment/Prime Type interaction	−0.018
	t = −1.963^*^
Containment/Question Type interaction	0.029
	t = 3.178^**^
Question Type/Prime Type interaction	−0.013
	t = −1.439
Three-way interaction	−0.007
	t = −0.722
Constant	7.663
	t = 246.117^***^
Observations	1,491
Log Likelihood	−613.253
Akaike Inf. Crit.	1,252.506
Bayesian Inf. Crit.	1,321.499

* p < 0.05;

** p < 0.01;

*** *p < 0.001*.

As in the previous experiment, we re-fitted the model using a treatment-contrast scheme in order to directly compare match and mismatch levels of the noun-question/animation-prime condition. This model showed no significant difference between these conditions, replicating the result found in Experiment 1 (see **Table 12**).

### 4.5. Discussion

In Experiment 2 we tried to facilitate the interaction between video prime and metaphoric verb by increasing statistical power and decreasing the temporal distance between verb and video. We again failed to find an effect of containment in the verb region. Besides this, we replicated the effect of prime type on all measures in the noun region: Seeing the word *opinion* written on the ball in the video facilitated reading times of that same word once it appeared in the sentence. This confirms that participants were able to use the information presented in the video to ease processing of the noun, and were nevertheless unable to use the feature of “containment” presented in the video to speed up (or slow-down) reading times in the verb region. This suggests that during processing of the metaphoric verb, participants largely ignored the feature of physical containment, seeing as it neither interfered with nor facilitated processing. This is consistent with a category inclusion view of metaphor comprehension that states that literal features are not initially activated if they are not necessary for the construction of the appropriate *ad hoc* category during metaphor processing.

However, it could also be the case that the lack of effects in the verb region is caused by inadequate materials: Activating the feature of spatial containment could indeed facilitate or hinder processing, but our video primes were simply not able to activate this feature. It is thus necessary to assess whether these videos could modulate processing in an environment in which they would be expected to do so reliably, namely when the verbs are processed in their encoded, literal meaning only. If the videos facilitate access to the literal meaning of the verbs, the current interpretation of the results of Experiments 1 and 2 becomes more plausible. We addressed this issue in Experiment 4.

The results of the question response task broadly replicated the findings of Experiment 1. It was easier for participants to answer the question in the match vs. mismatch levels of the video-question conditions. In the noun-question conditions, there was an effect of prime type, with the animation-prime conditions showing slower response times than the mixed-prime conditions.

The noun-question/animation-prime conditions did not show a significant difference between match and mismatch levels, just as in Experiment 1. This finding is important because the noun-question/animation-prime conditions were the only ones without a confound between condition and correct answer. Furthermore, there was an effect of prime type in the noun-question conditions, with the “animation” conditions showing longer response times than the “mixed” conditions.

As mentioned in the discussion of Experiment 1, these results could be interpreted as meaning that when reading the sentence, the conceptual feature of containment is activated, facilitating responses in the match vs. mismatch conditions and interfering with the responses in the noun-question/animation-prime conditions.

This interpretation, however, is contingent upon the assumption that the response patterns were caused by the interaction of processing video and metaphor and not by the YES/NO response confound or by other external factors. We sought to test this assumption in Experiment 3.

## 5. Experiment 3

Question-response times in Experiments 1 and 2 show an overall facilitation effect for match vs. mismatch conditions, except for the noun-question/animation-prime conditions, which showed no difference between match and mismatch levels. In Experiment 3, we set out to test whether these results were caused by the interaction of video, metaphor and question, or whether they could be explained by the interaction of video and question only. To do this, we ran a version of Experiment 2 in which the sentences read by participants did not contain any metaphors whatsoever: If the same pattern of results as in the previous two experiments is visible, it would suggest that the results are not related to the processing of verbal metaphors. Since we were not interested in the reading patterns of these sentences, but only in the question-response times, Experiment 3 was not run as an eye-tracking study. Instead, it was implemented as a self-paced reading reaction time task: Participants first watched the video-prime and then read the (non-metaphoric) sentence. When they were done reading, they pushed a button in front of them and were presented with the comprehension questions, which they answered by pushing either a YES or NO button. We measured only the response times to the comprehension questions.

### 5.1. Participants

Sixty-four native speakers of German (ages 18–31, 34 female) with normal or corrected-to-normal vision were recruited and tested at the Humboldt-Universität zu Berlin. None of them had participated in Experiments 1, or 2. They gave their informed consent and received 8 euros as compensation after completing the experiment. Experiment 3 was covered by the ethics vote granted to the psycholinguistics lab of the Humboldt-Universität zu Berlin by the German Linguistic Society (Deutsche Gesellschaft für Sprachwissenschaft, DGfS).

### 5.2. Materials and Design

To construct the materials in Experiment 3, we modified the sentences from Experiment 2 by replacing the verb with a non-metaphorical one that did not have the feature of spatial containment as part of its literal meaning, as presented in (5):

(5) “*Dass seine Meinung ignoriert wurde nach dem Regimewechsel, war für den Redakteur schwierig”*
  “The fact that his opinion was ignored after the change in regime was difficult for the journalist”

The design was identical to that of the previous experiments, with the factors containment, question type and prime type. The experiment was programmed using the open source software Open Sesame and was run on a PC computer. The only dependent measure in this experiment was question response time.

### 5.3. Procedure

Participants were instructed to wear noise-reducing headphones throughout the experiment to avoid being distracted by the other participants. Each trial consisted of three phases: First, participants saw the same animated video presented in experiments 1–3. They then read a sentence and pressed the space bar on the keyboards that was in front of them. The sentence then disappeared and a question appeared on the screen. They had to answer this question by pressing either the letter F or J, which were counterbalanced across participants to stand for either YES or NO.

### 5.4. Predictions

Our predictions are derived from the results of Experiments 1 and 2: If we find the same pattern of results in Experiment 3 as in the previous two iterations, it would suggest that the results were not driven by the interaction of video, metaphor and question, but just by the interaction of video and question, given that there are no metaphors in Experiment 3. If we find a different pattern than this, it would suggest that the results found in Experiments 1 and 2 were (at least partially) caused by the way participants processed the verbal metaphors. In this sense, Experiment 3 serves as a baseline against which we can interpret the results of the question-response times of Experiments 1 and 2. Of particular interest are again the noun-question/animation-prime conditions: These are the only match/mismatch pair where both the question asked and the correct response remained constant.

### 5.5. Results

**Figure 11:**
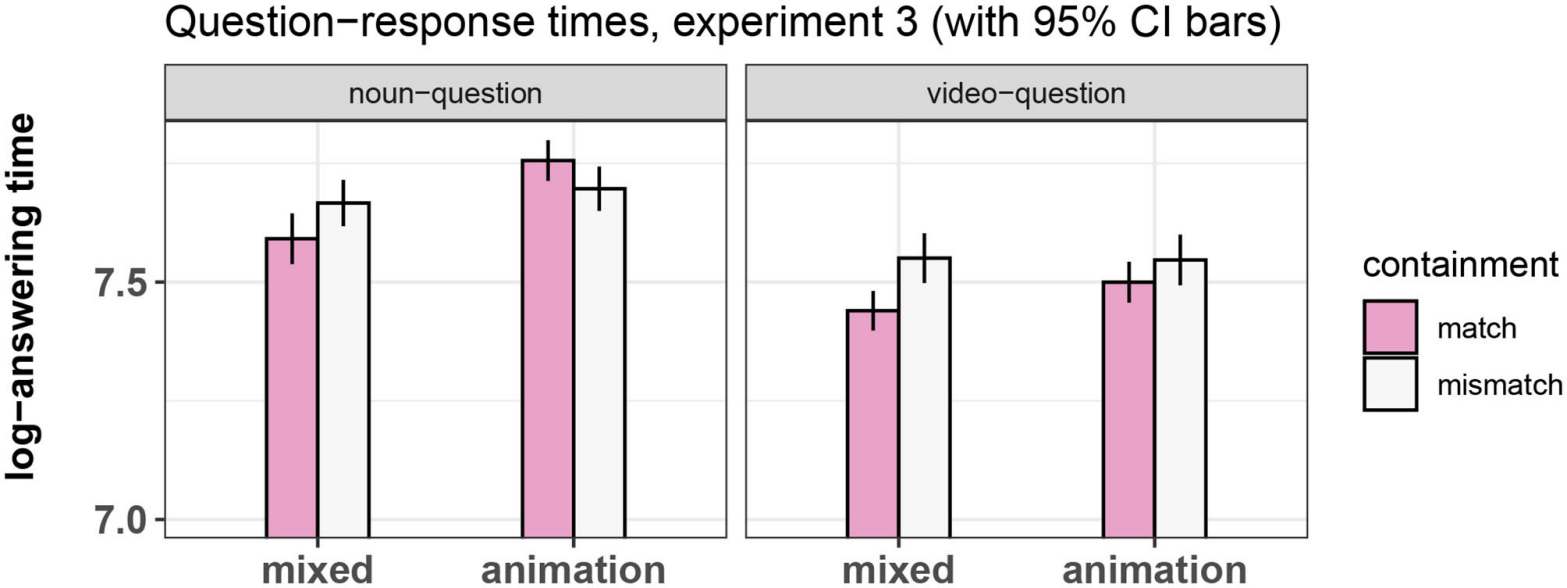
Summary of results for the question response time, Experiment 3.

We fitted a linear mixed effects regression model to the log-transformed reaction times. We found a main effect of containment, prime type and question type. We also found significant interactions of containment and question type, containment and prime type, question type and prime type and question type, prime type and containment. The results are shown in Figure 11 and the model details are given in Table 11.

**Table 11:** Regression analysis of response-times in Experiment 3.

	**Dependent variable**
	**Response times (in log-milliseconds)**
Containment	−0.043
	t = −2.281^*^
Prime Type	0.168
	t = 9.631^***^
Question Type	−0.062
	t = −3.881^***^
Trial order	−0.001
	t = −1.307
Containment/Prime Type interaction	0.071
	t = 2.219^*^
Containment/Question Type interaction	−0.099
	t = −2.936^**^
Question Type/Prime Type interaction	−0.067
	t = −2.105^*^
Three-way interaction	−0.069
	t = −1.088
Constant	7.612
	t = 466.819^***^
Observations	2,113
Log Likelihood	−887.236
Akaike Inf. Crit.	1,822.472
Bayesian Inf. Crit.	1,958.213

* p < 0.05;

** p < 0.01;

*** *p < 0.001*.

Re-fitting the model with treatment contrasts, as we did for the previous experiments, showed a significant difference between match and mismatch levels of the noun-question/animation-prime conditions, with the match condition showing significantly faster responses than the mismatch condition. The details of this model are shown in Table 12.

**Table 12:** Model fitted with treatment-contrast coding for response times of Experiments 1–3.

	**Dependent variable**		
	**Response times per Experiment (in log-milliseconds)**		
	**(1)**	**(2)**	**(3)**
Containment	−0.066	−0.074	−0.064
	t = −1.436	t = −1.803	t = −2.185^*^
Constant	7.506	7.913	7.947
	t = 162.766^***^	t = 188.551^***^	t = 235.400^***^
Observations	1,111	1,491	2,113
Log Likelihood	−559.837	−602.232	−549.023
Akaike Inf. Crit.	1,145.674	1,256.464	1,150.046
Bayesian Inf. Crit.	1,210.843	1,394.452	1,297.098

* p < 0.05;

^**^p < 0.01;

*** *p < 0.001*.

*“Containment” shows effect in noun-question/animation-prime conditions only*.

### 5.6. Discussion

The pattern of results is very similar to that found in Experiments 1 and 2. This suggests that the response times found in those experiments were mostly modulated by factors independent of the metaphorical verb, since there was no metaphorical verb in Experiment 3. This confirms the simple explanation that the response time results follow from a general response bias (Easier to answer YES than NO and easier to answer to shorter than to longer questions), and are not a product of metaphoric interpretation.

However, the results of the noun-question/animation-prime conditions require further explanation. In Experiment 3, the match vs. mismatch conditions were significantly different from one another, whereas in Experiments 1 and 2, no significant difference was found. It is thus likely that this difference between experiments is the only one that is contingent on the presence of the metaphorical sentences in Experiments 1 and 2: If in the absence of a metaphor there are shorter response times in the mismatch compared to the match level of the noun-question/animation-prime condition (our baseline result), then the lack of a difference between conditions in the presence of a metaphor (Experiments 1 and 2) could actually be interpreted as a facilitation effect of the match compared to the mismatch condition relative to the baseline result of Experiment 3.

This interpretation, as well as the interpretation of the results of the gaze record of Experiments 1 and 2, relies on the assumption that participants can indeed derive the conceptual feature of containment from our prime videos and that this feature interacts with the way the verbs are processed. Experiment 4 directly addresses this issue.

## 6. Experiment 4

In this experiment we dealt with the question of whether or not the videos used in Experiments 1–3 can activate a mental representation of containment that leads participants to process verbs of physical containment more readily than when they first see a video that does not depict containment.

### 6.1. Participants

A sample of 259 German native speakers (ages 18–31, 120 female) were recruited online via the platform “clickworker.” They gave their informed consent and received 50 cents as compensation upon finishing the experiment. Experiment 4 was covered by the data protection policy of the Humboldt-Universität zu Berlin.

### 6.2. Materials and Design

Experiment 4 was a web-based lexical decision task in which participants saw the same video clips from Experiments 1–3 as primes and then read the same verbs from Experiments 1 and 2, which were presented here without context. The experiment thus only had the factor containment with the levels match and mismatch.

### 6.3. Procedure

The experiment was designed and run using an instance of the IBEX farm (created by Alex Drummond) coupled with the Penncontroller extension (Zehr and Schwarz, 2018), which allows for a simple integration of video and linguistic stimuli. On each trial, participants first saw a video prime and then a target word in the middle of the screen, and had a total of 5 s to decide whether the word was a real word by either pressing F (“not a real word”) or J (“real word”). After one practice item, participants were presented with six experimental trials (two critical, four fillers). There was a 1 s pause in-between trials. One experimental session lasted around 4–5 min.

### 6.4. Predictions

If the video in the “match” condition is not capable of eliciting a mental representation of “containment” that can aid lexical recognition of verbs of physical containment, there should be no difference in reaction times between conditions. If, on the other hand, the video in the “match” condition is indeed capable of eliciting a mental representation of “containment” that can ease lexical recognition of verbs of physical containment, we expect shorter reaction times in the match condition compared to the mismatch condition.

### 6.5. Analysis and Results

Prior to the analysis, participants who got <4/6 correct responses were excluded (*n* = 9), leaving the total number of participants at 250. Reaction times were log-transformed following the results of a box-cox test (Box and Cox, 1964).

**Figure 12:**
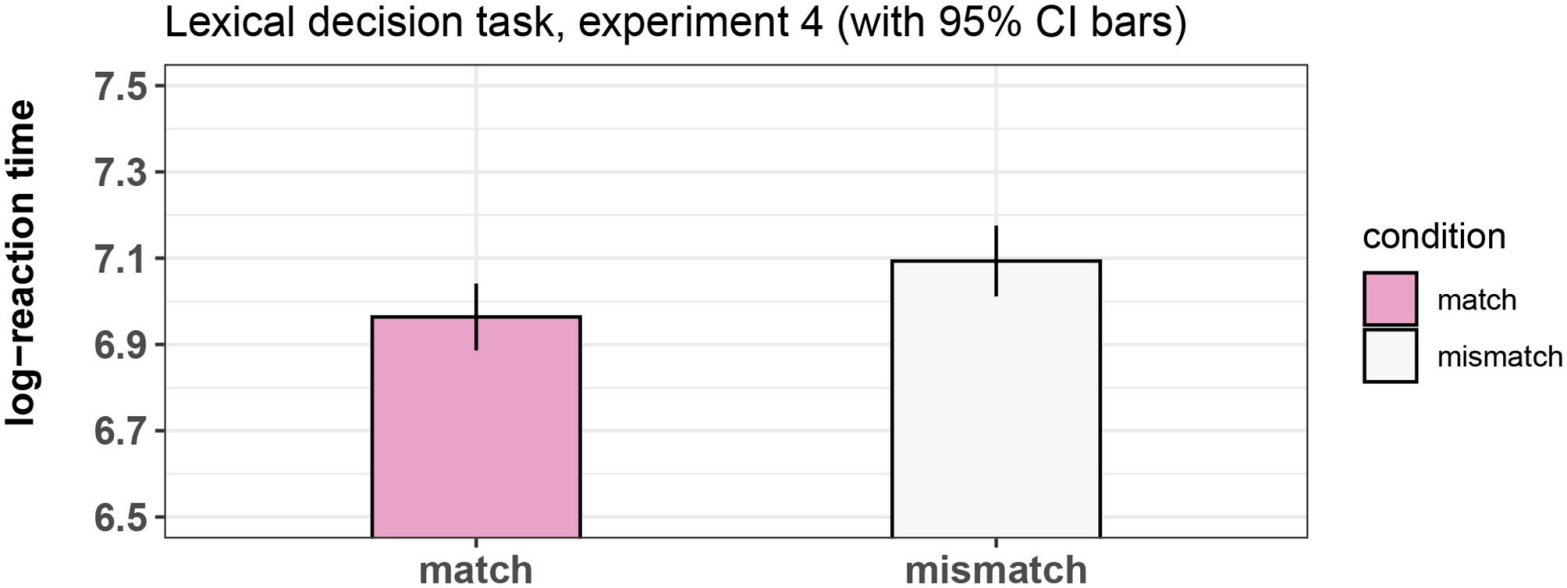
Summary of results for the lexical decision task, Experiment 4.

A linear mixed effects model was then fitted to the data. The results showed a significant difference between the two conditions, with the match condition displaying shorter reaction times compared to the mismatch condition. The effect size had a value of Cohen's *d* = 0.21 (i.e., a “small” effect size according to Cohen, 1992). The results are presented in Figure 12 and the model summary in Table 13.

**Table 13:** Regression analysis of response-times in Experiment 4.

	**Dependent variable**
	**Response times (in log-milliseconds)**
Containment	0.133
	t = 2.993^**^
Constant	6.974
	t = 153.213^***^
Observations	465
Log Likelihood	−415.286
Akaike Inf. Crit.	840.571
Bayesian Inf. Crit.	861.282

^*^p < 0.05;

** p < 0.01;

*** *p < 0.001*.

### 6.6. Discussion

Experiment 4 showed that the video-clip primes used in Experiments 1–3 facilitated the retrieval of the encoded, literal meaning of different verbs of physical containment. This finding suggests that participants were able to derive the conceptual feature of physical containment from the videos in the match conditions, since this is the key feature we believe the videos share with the verbs.

## 7. General Discussion and Conclusion

Theories of metaphor processing make different predictions regarding the role of conceptual features related only to the literal meaning during and immediately after processing of (novel) metaphors. Category inclusion views believe that these literal features should not play a role during processing and might even hinder comprehension (McGlone and Manfredi, 2001). Furthermore, they should be rapidly suppressed after the metaphor has been understood (Gernsbacher et al., 2001; Rubio Fernandez, 2007). Indirect comparison views, instead, claim that features related to the literal meaning of a metaphor are initially active. This is caused by an alignment stage in which encoded meanings are fully retrieved prior to the projection of inferences (Gentner et al., 2001; Bowdle and Gentner, 2005, i.a.). This means that literal features should facilitate early stages of processing, as shown by Weiland et al. (2014), and can remain active after comprehension, easing understanding of subsequent, related novel, or conventional metaphors (Thibodeau and Durgin, 2008).

In our investigation, we looked at how priming the conceptual feature of spatial containment would interact with the processing of verbal metaphors in which physical containment is a crucial part of the literal meaning but (arguably) not of the metaphoric interpretation. The results of two eye-tracking experiments showed that the videos neither facilitated nor hindered processing of the verbs used (e.g., *fenced-in*), regardless of whether the verb appeared early on or late in the sentence (Experiments 1 and 2). This absence of an effect was accompanied by a reliable priming effect of the noun that appeared in both video and sentence, suggesting that participants were actively integrating the input of the video with the input of the sentence. Furthermore, we showed that the videos did elicit a priming effect on those same verbs in a de-contextualized lexical decision task (Experiment 4).

Data from the question-response times showed that participants were overall faster answering questions in the match vs. mismatch conditions. They were also overall slower to answer questions about the interaction between video and sentence (*Was the opinion in the box?*) than about just the video. Since these effects were present in both the experiments with a metaphoric verb (Experiments 1 and 2) and our baseline experiment without a metaphoric verb (Experiment 3) they do not tell us much about how the metaphors interacted with video and question type during processing. However, in the absence of a metaphor (Experiment 3), participants were significantly faster at correctly answering the question in the noun-question/animation-prime mismatch condition (*Was the opinion in the box?* When there was no word written on the ball and the ball bounced freely) compared to the noun-question/animation-prime match condition (*Was the opinion in the box?* When there was no word written on the ball and the ball was trapped by the box). In Experiments 1 and 2, there was no difference between these conditions. This suggests that in the presence of a metaphor there could be a facilitation effect of the match compared to the mismatch noun-question/animation-prime conditions, which might mean that the metaphor itself activated the feature of spatial containment which later facilitated response times to the post-sentence questions. However, the evidence for this is very tenuous since the overall question-response pattern in all three experiments was similar.

We interpret the data as showing that the feature of physical containment is ignored during comprehension of novel verbal metaphors of containment and neither facilitates nor hinders processing. Failing to find a significant difference between conditions is not equivalent to finding that there is no difference between them. However, given the results of Experiment 4 and the fact that in Experiments 1 and 2 there was a significant effect of prime type (showing that some aspects of the prime were indeed integrated with the sentence), we believe that the absence of an effect of containment in Experiments 1 and 2 can be interpreted as meaningful.

We see this as being in line with a metaphor processing view that does not ascribe an important role to literal features of the metaphoric vehicle during initial stages of processing. Such is the case of category membership views (Glucksberg, 2001; Sperber and Wilson, 2008), which claim that the meaning of the vehicle is quickly modulated given the dimensions provided by the topic. In this process, features of the literal meaning that are not compatible with the dimensions provided by the topic do not need to be activated. However, pre-activating these features does not interfere with the lexical modulation of the metaphoric vehicle either.

It is important to note that the goal of the current set of studies was to investigate novel verbal metaphors only. Given that other factors, such as conventionality (Bowdle and Gentner, 2005), aptness (Jones and Estes, 2006), and familiarity (Thibodeau and Durgin, 2011) can modulate metaphor processing, it would be interesting to observe whether the current results would hold when examining metaphors that varied along those three dimensions. We leave this specific point for future research to examine.

Furthermore, it could be that metaphor processing varies according to syntactic class such that nominal metaphors are processed differently than verbal metaphors. This would mean that nominal metaphors could be understood via indirect comparison (following Gentner and Bowdle, 2008) and verbal metaphors via lexical modulation (as posited by category inclusion views). However, neuroimaging evidence suggests that the mechanisms for different types of metaphors might be the same. Cardillo et al. (2012) investigated processing of both nominal and verbal metaphors using functional magnetic resonance. Their results show that the neural processes associated with both of these types of metaphors do not differ significantly, suggesting that the underlying cognitive mechanisms are likely the same. We therefore believe that our results generalize beyond the case of verbal metaphors.

In terms of how our results relate to the literature on the interaction between language and the visual world we can draw the following conclusions: Guerra and Knoeferle (2014) found a facilitation effect of visual primes of distance on processing of semantic similarity. They argued that this was indicative of an abstract co-indexing link between distance and similarity. In Experiments 1 and 2 of the current investigation we failed to find such a link between videos of containment and adjectives of difficulty. It could be the case that these co-indexing links are constructed and stored in memory via repeated, conventional use: Perhaps speaking of semantic similarity in terms of distance is a more common occurrence than speaking of difficulty in terms of containment, leading to facilitation effects in the former but not in the latter case.

In a production study, Sato et al. (2015) found a priming effect of metaphors of difficulty after participants saw images of physical containment, an effect which we failed to find in the present language comprehension study. This difference in results could be explained by a difference in conventionality of the types of metaphors used: Sato et al. (2015) counted the production of spatial prepositions, such as *in* and *out* (e.g., *Bobbie fell in love working in the potato factory*) and of idiomatic expressions (*Nick said time is full of shit*) as instances of a containment-as-difficulty metaphor. These types of conventional, “fossilized” metaphoric expressions are likely to be processed differently than novel metaphors (Keysar et al., 2000; Bowdle and Gentner, 2005) making the results difficult to compare, given that the materials in our study were all novel verbal metaphors (It is not clear whether participants in the study by Sato and collaborators even produced any novel metaphors at all).

There are some caveats with our interpretation of the results: First, in Experiment 4 each participant saw only two critical items, whereas in Experiments 1 and 2 participants saw the full set of 36 items. It could therefore be the case that repeated exposure to the video primes interfered with an underlying true priming effect that our experimental set-up in Experiments 1 and 2 could not detect. To assess this possibility we conducted *post-hoc* analyses examining the pattern of results of Experiments 1 and 2 in the first third of the Experiment (i.e., after 36 trials). These showed the exact same pattern found for the entire experiment (i.e., no effect of video-prime on reading measures). It is thus not likely that a repetition effect is solely responsible for the differences in effect found between Experiments 1, 2, and 4.

It is also possible that the lack of an effect was due to the verbs being embedded in a sentence, regardless of whether the context encourages a literal or metaphoric interpretation of the verb. This is unlikely, considering that in Experiment 2 the Video-Prime and the verb were almost as temporally adjacent as in Experiment 4, but it cannot be ruled out completely. Further research is necessary in order to determine the exact nature of the prime-verb relation and the different contexts under which a priming effect could arise. We nevertheless see our set of experiments as a step forward in understanding how metaphors are processed outside of the narrow realm of nominal metaphors.

## Data Availability Statement

The raw data supporting the conclusions of this article will be made available by the authors, without undue reservation.

## Ethics Statement

The studies involving human participants were reviewed and approved by Ethics committee of the Humboldt-Universität zu Berlin. The patients/participants provided their written informed consent to participate in this study.

## Author Contributions

CR, EG, and PK planned and designed the experiments, edited the paper, and produced the final version. CR conducted the experiments and wrote the first draft of the paper. CR and EG analyzed the data. All authors contributed to the article and approved the submitted version.

## Conflict of Interest

The authors declare that the research was conducted in the absence of any commercial or financial relationships that could be construed as a potential conflict of interest.

## Footnotes

^1^Whether aptness or conventionality modulates the processing route is still a matter of debate and outside of the scope of the current investigation, which focuses on novel verbal metaphors only. For in-depth discussions on the role these factors might play during processing see Gentner and Bowdle (2008), Glucksberg (2008), Holyoak and Stamenković (2018), and Pouscoulous and Dulcinati (2019).

## Supplementary Material

Video 1 Example prime video for a critical item in the containment-mismatch conditions used in Experiments 1–4 (no text was written on the ball in Experiments 1 and 4).

Video 2 Example prime video for a critical item in the containment-match conditions used in Experiments 1–4 (no text was written on the ball in Experiments 1 and 4).
