# Supplementary material for: The Role of Literal Features During Processing of Novel Verbal Metaphors
Source: Front Psychol. 2021 Jan 26;11:556624. doi: 10.3389/fpsyg.2020.556624 (PMC7870694; doi:10.3389/fpsyg.2020.556624)
Supplement: Supplementary file 3 [file Data_Sheet_1.pdf]

Supplementary materials
Verbal metaphors

A: Critical sentences for experiment 1
B: Critical sentenecs for experiment 2
C: Critical sentences for experiment 3
D: Target words for Experiment 4
E: Filler sentences for experiments 1-3

A

1. Es war für den Polizisten schwierig, seinen Aufstiegsdrang nach dem Korruptionsvorwurf gedeckelt zu sehen.
2. Es war für den Auszubildenden schwierig, seine Ambitionen nach dem Rechenfehler gedeckelt zu sehen.
3. Es war für den Premierminister schwierig, seinen Einfluss nach der Wahlschlappe ummauert zu sehen.
4. Es war für den Gruppenleiter schwierig, seine Unabhängigkeit nach dem Betrugsfall ummauert zu sehen.
5. Es war für den Moderator schwierig, seine Experimentierfreude nach dem Quotensturz gefesselt zu sehen.
6. Es war für den Arzt schwierig, seine Ermessensfreiheit nach der Fehleinschätzung umrandet zu sehen.
7. Es war für den Rentner schwierig, seine Selbstständigkeit nach der Diagnose umrandet zu sehen.
8. Es war für den Schüler schwierig, seine Freizeit nach der Zeugnisvergabe umwickelt zu sehen.
9. Es war für den Stürmer schwierig, seine Trainingszeit nach der Verletzung umgrenzt zu sehen.
10. Es war für den Studenten schwierig, sein Mitbestimmungsrecht nach den Protesten umgrenzt zu sehen.
11. Es war für den Soldaten schwierig, seinen Optimismus nach der Niederlage umgittert zu sehen.
12. Es war für den Redakteur schwierig, seine Meinung nach dem Regimewechsel umgittert zu sehen.
13. Es war für den Staranwalt schwierig, seine Siegesgewissheit nach der Beweisaufnahme umzäunt zu sehen.
14. Es war für den Lehrer schwierig, seine Privilegien nach der Bildungsreform umzäunt zu sehen.
15. Es war für den Bildhauer schwierig, seine Entwicklung nach der Akademieschließung beengt zu sehen.
16. Es war für den Helden schwierig, seine Kraft nach dem Verkehrsunfall beengt zu sehen.
17. Es war für den Investor schwierig, seine Expansionspläne nach dem Börsenkrach eingeschnürt zu sehen.
18. Es war für den Musiker schwierig, seinen Freigeist nach der Konzertpleite eingeschnürt zu sehen.

19. Es war für den Grundbesitzer schwierig, seine Kauflust nach der Revolution eingekapselt zu sehen.
20. Es war für den Künstler schwierig, seine Gestaltungsfreiheit nach der Zeitungskritik eingekapselt zu sehen.
21. Es war für den Priester schwierig, seine Autonomie nach den Skandalen einbetoniert zu sehen.
22. Es war für den General schwierig, seine Befugnisse nach dem Militärputsch einbetoniert zu sehen.
23. Es war für den Abgeordneten schwierig, seinen Gesetzentwurf nach der Staatskrise eingeklemmt zu sehen.
24. Es war für den Professor schwierig, sein Forschungsvorhaben nach der Umstrukturierung eingeklemmt zu sehen.
25. Es war für den Opernsänger schwierig, sein Selbstvertrauen nach dem Saison-Fehlstart festgekettet zu sehen.
26. Es war für den Architekten schwierig, seine Schöpferkraft nach der Budgetkürzung festgekettet zu sehen.
27. Es war für den Anleger schwierig, seine Risikofreude nach dem Kurssturz festgebunden zu sehen.
28. Es war für den König schwierig, seine Macht nach der Verfassungsänderung festgebunden zu sehen.
29. Es war für den Vermieter schwierig, seine Ansprüche nach der Mietrechtsreform abgesteckt zu sehen.
30. Es war für den Unternehmensleiter schwierig, sein Vermächtnis nach der Verstaatlichung abgesteckt zu sehen.
31. Es war für den Clown schwierig, seinen Humor nach der Panne eingedämmt zu sehen.
32. Es war für den Ganoven schwierig, seine Überlegenheit nach der Razzia eingedämmt zu sehen.
33. Es war für den Reporter schwierig, seine Recherchemöglichkeiten nach dem Berufsverbot abgeriegelt zu sehen.
34. Es war für den Bauern schwierig, seine Förderungschancen nach der Gesetzesänderung abgeriegelt zu sehen.
35. Es war für den Bürgermeister schwierig, seine Karriere nach dem Misstrauensantrag abgesperrt zu sehen.
36. Es war für den Oppositionellen schwierig, seine Zuversicht nach der Parteispaltung abgesperrt zu sehen.

B

1. Dass sein Aufstiegsdrang gedeckelt wurde nach dem Korruptionsvorwurf war schwierig für den Polizisten.
2. Dass seine Ambitionen gedeckelt wurden nach dem Rechenfehler war schwierig für den Auszubildenden.
3. Dass sein Einfluss ummauert wurde nach der Wahlschlappe war schwierig für den Premierminister.
4. Dass seine Unabhängigkeit ummauert wurde nach dem Betrugsfall war schwierig für den Gruppenleiter.

5. Dass seine Experimentierfreude gefesselt wurde nach dem Quotensturz war schwierig für den Moderator.
6. Dass seine Ermessensfreiheit umrandet wurde nach der Fehleinschätzung war schwierig für den Arzt.
7. Dass seine Selbstständigkeit umrandet wurde nach der Diagnose war schwierig für den Rentner.
8. Dass seine Freizeit umwickelt wurde nach der Zeugnisvergabe war schwierig für den Schüler.
9. Dass seine Trainingszeit umgrenzt wurde nach der Verletzung was schwierig für den Stürmer.
10. Dass sein Mitbestimmungsrecht umgrenzt wurde nach den Protesten war schwierig für den Studenten.
11. Dass sein Optimismus umgittert wurde nach der Niederlage war schwierig für den Soldaten.
12. Dass seine Meinung umgittert wurde nach dem Regimewechsel war schwierig für den Redakteur.
13. Dass seine Siegesgewissheit umzäunt wurde nach der Beweisaufnahme war schwierig für den Staranwalt.
14. Dass seine Privilegien umzäunt wurden nach der Bildungsreform war schwierig für den Lehrer.
15. Dass seine Entwicklung beengt wurde nach der Akademieschließung war schwierig für den Bildhauer.
16. Dass seine Kraft beengt wurde nach dem Verkehrsunfall war schwierig für den Helden.
17. Dass seine Expansionspläne eingeschnürt wurden nach dem Börsenkrach war schwierig für den Investor.
18. Dass sein Freigeist eingeschnürt wurde nach der Konzertpleite war schwierig für den Musiker.
19. Dass seine Kauflust eingekapselt wurde nach der Revolution war schwierig für den Grundbesitzer.
20. Dass seine Gestaltungsfreiheit eingekapselt wurde nach der Zeitungskritik war für den Künstler schwierig.
21. Dass seine Autonomie einbetoniert wurde nach den Skandalen war schwierig für den Priester.
22. Dass seine Befugnisse einbetoniert wurden nach dem Militärputsch war schwierig für den General.
23. Dass sein Gesetzentwurf eingeklemmt wurde nach der Staatskrise war für den Abgeordneten schwierig.
24. Dass sein Forschungsvorhaben eingeklemmt wurde nach der Umstrukturierung war schwierig für den Professor.
25. Dass sein Selbstvertrauen festgekettet wurde nach dem Saison-Fehlstart war schwierig für den Opernsänger.
26. Dass seine Schöpferkraft festgekettet wurde nach der Budgetkürzung war schwierig für den Architekten.
27. Dass seine Risikofreude festgebunden wurde nach dem Kurssturz war schwierig für den Anleger.
28. Dass seine Macht festgebunden wurde nach der Verfassungsänderung war schwierig für den König.

29. Dass seine Ansprüche abgesteckt wurden nach der Mietrechtsreform war schwierig für den Vermieter.
30. Dass sein Vermächtnis abgesteckt wurde nach der Verstaatlichung war schwierig für den Unternehmensleiter.
31. Dass sein Humor eingedämmt wurde nach der Panne war schwierig für den Clown.
32. Dass seine Überlegenheit eingedämmt wurde nach der Razzia war schwierig für den Ganoven.
33. Dass seine Recherchemöglichkeiten abgeriegelt wurden nach dem Berufsverbot war schwierig für den Reporter.
34. Dass seine Förderungschancen abgeriegelt wurden nach der Gesetzesänderung war schwierig für den Bauern.
35. Dass seine Karriere abgesperrt wurde nach dem Misstrauensantrag war schwierig für den Bürgermeister.
36. Dass seine Zuversicht abgesperrt wurde nach der Parteispaltung war schwierig für den Oppositionellen.

C

1. Dass sein Aufstiegsdrang beneidet wurde nach dem Korruptionsvorwurf, war schwierig für den Polizisten.
2. Dass seine Ambitionen beneidet wurden nach dem Rechenfehler, war schwierig für den Auszubildenden.
3. Dass sein Einfluss unterschätzt wurde nach der Wahlschlappe, war schwierig für den Premierminister.
4. Dass seine Unabhängigkeit erörtert wurde nach dem Betrugsfall, war schwierig für den Gruppenleiter.
5. Dass seine Experimentierfreude befürchtet wurde nach dem Quotensturz, war schwierig für den Moderator.
6. Dass seine Ermessensfreiheit erörtert wurde nach der Fehleinschätzung, war schwierig für den Arzt.
7. Dass seine Selbstständigkeit angezweifelt wurde nach der Diagnose, war schwierig für den Rentner.
8. Dass seine Freizeit gekürzt wurde nach der Zeugnisvergabe, war schwierig für den Schüler.
9. Dass seine Trainingszeit gekürzt wurde nach der Verletzung was schwierig für den Stürmer.
10. Dass sein Mitbestimmungsrecht aberkannt wurde nach den Protesten, war schwierig für den Studenten.
11. Dass sein Optimismus bemitleidet wurde nach der Niederlage, war schwierig für den Soldaten.
12. Dass seine Meinung ignoriert wurde nach dem Regimewechsel, war schwierig für den Redakteur.
13. Dass seine Siegesgewissheit überrumpelt wurde nach der Beweisaufnahme, war schwierig für den Staranwalt.
14. Dass seine Privilegien beklagt wurden nach der Bildungsreform, war schwierig für den Lehrer.
15. Dass seine Entwicklung bewundert wurde nach der Akademieschließung, war schwierig für den Bildhauer.

16. Dass seine Kraft bewundert wurde nach dem Verkehrsunfall, war schwierig für den Helden.
17. Dass seine Expansionspläne aufgeschoben wurden nach dem Börsenkrach, war schwierig für den Investor.
18. Dass sein Freigeist beleidigt wurde nach der Konzertpleite, war schwierig für den Musiker.
19. Dass seiner Kauflust misstraut wurde nach der Revolution, war schwierig für den Grundbesitzer.
20. Dass seine Gestaltungsfreiheit beleidigt wurde nach der Zeitungskritik, war für den Künstler schwierig.
21. Dass seine Autonomie diskutiert wurde nach den Skandalen, war schwierig für den Priester.
22. Dass seine Befugnisse ignoriert wurden nach dem Militärputsch, war schwierig für den General.
23. Dass sein Gesetzentwurf aufgeschoben wurde nach der Staatskrise, war für den Abgeordneten schwierig.
24. Dass sein Forschungsvorhaben thematisiert wurde nach der Umstrukturierung, war schwierig für den Professor.
25. Dass sein Selbstvertrauen überrumpelt wurde nach dem Saison-Fehlstart, war schwierig für den Opernsänger.
26. Dass seine Schöpferkraft angezweifelt wurde nach der Budgetkürzung, war schwierig für den Architekten.
27. Dass seine Risikofreude thematisiert wurde nach dem Kurssturz, war schwierig für den Anleger.
28. Dass seine Macht belächelt wurde nach der Verfassungsänderung, war schwierig für den König.
29. Dass seine Ansprüche diskutiert wurden nach der Mietrechtsreform, war schwierig für den Vermieter.
30. Dass sein Vermächtnis besteuert wurde nach der Verstaatlichung, war schwierig für den Unternehmensleiter.
31. Dass sein Humor bemitleidet wurde nach der Panne, war schwierig für den Clown.
32. Dass seine Überlegenheit unterschätzt wurde nach der Razzia, war schwierig für den Ganoven.
33. Dass seinen Recherchemöglichkeiten misstraut wurde nach dem Berufsverbot, war schwierig für den Reporter.
34. Dass seine Förderungschancen benachteiligt wurden nach der Gesetzesänderung, war schwierig für den Bauern.
35. Dass seine Karriere beendet wurde nach dem Misstrauensantrag, war schwierig für den Bürgermeister.
36. Dass seine Zuversicht belächelt wurde nach der Parteispaltung, war schwierig für den Oppositionellen.

D

1. gedeckelt
2. beengt
3. ummauert
4. gefesselt
5. umrandet

6. umwickelt
7. umgrenzt
8. umgittert
9. umzäunt
10. eingeschnürt
11. eingekapselt
12. einbetoniert
13. eingeklemmt
14. festgekettet
15. festgebunden
16. abgesteckt
17. abgeriegelt
18. abgesperrt

E

1. Köstlichkeit und Schokolade sind absolut identisch, so meinte das Mädchen.
2. Vollbeschäftigung und Spaß am Leben scheinen nicht zusammenzupassen, bedauerte die Apothekerin.
3. Betrübnis und Euphorie sind Erscheinungen der Krankheit, das bedauerte der Patient.
4. Streiterei und Zwietracht sind uns neuerdings eine Last geworden, das beklagte die Priesterin.
5. Antipathie und Liebelei sind zwei Seiten einer Medaille, das stellte der Schreiber fest.
6. Arbeitszeit und Vergnügen ergänzen sich gegenseitig, das suggerierte der Tennis-Profi.
7. Erniedrigung und Entwürdigung sind genauso furchtbar, das verteidigte die Soziologin.
8. Fairness und Mogelei sind sicher Teil des Spiels, das weissagte der Schiedsrichter.
9. Dissonanz und Wohlklang sind sicher ein Teil meiner sehr großen Werkzeugkiste, das bejahte der Gitarrist.
10. Arbeitsverträge und Lesevergnügen sind doch dasselbe, schreit die Beraterin ironisch.
11. Nüchternheit und Pünktlichkeit sehen anders aus, erwiderte die Gattin als der Herzog nach Hause kam.
12. Pistazien und Erdnüsse schmecken bestimmt gleich, das behauptete die Bäckerin.
13. Schlafen und Arbeiten gehören nicht zusammen, ermahnte die Rettungssanitäterin ihren Kollegen.
14. Verschwörung und Veruntreuung sind eher eine Frage der Perspektive, das gab die Präsidentin bekannt.
15. Anlächeln und flirten sind doch nicht dasselbe, meinte das Model zu ihrem wütenden Freund.
16. Apple und Windows sind gar nicht identisch, widersprach der genervte Nerd.
17. Vergötterung und Nichtbeachtung sind heute gang und gäbe, das beklagte die Nonne.
18. Trockenheit und Sauberkeit sind Ihnen gewiß fremd, brüllte Karla zu dem Klempner.
19. Feinkost und Italien sind bestimmt gleichbedeutend, das betonte der Tourist.
20. Langeweile und Fernsehen sind ein und dasselbe, so beurteilte die Mutter.

21. Gereiztheit und Trinkgeld gehen nie Hand in Hand, so scherzte die Kellnerin.
22. Religion und Gehirnwäsche liegen dicht beieinander, so schreit die Erzieherin.
23. Geduld und Fleiß sind ähnlich wichtig für den Erfolg, das erzählte die Malerin.
24. Arbeitslosigkeit und Suff passen ganz gut zusammen, so rechtfertigte sich der Alkoholiker.
25. Dass die Republikaner im siebten Himmel schwebten nach der Wahl war nervig für den Prinzen.
26. Das A und O der Widerstandsbewegung zu kennen war wichtig für den Senator.
27. Sich nicht von dem Senat die Butter vom Brot nehmen zu lassen war für den Berliner wichtig.
28. Es war für den Diktator wichtig, vor dem Attentat den Braten noch gerochen zu haben.
29. Es war für die Mutter traurig, immer nach dem zweiten Glas Wein die Sau rauslassen zu müssen.
30. Dass er von seinem Partner in die Pfanne gehauen wurde, war für den Einzelhändler traurig.
31. Dass er während der Budgetplanung am Katzentisch sitzen musste, war traurig für den Sozialarbeiter.
32. Dass der Herrscher nicht alle Tassen im Schrank hatte, war traurig für die Bürger.
33. Dass die kleine Stadt auf den Hund gekommen ist, war traurig für Thomas.
34. Dass der Tutor immer auf dem Schlauch stand, war für die Dozentin nervig.
35. Dass sie sich die Gehaltserhöhung durch die Lappen gehen ließ, war nervig für die Druckerin.
36. Dass sie sich während des Streiks vom Acker gemacht hatte, war für die Firmeninhaberin selbstverständlich.
37. Dass ihre Kunden immer das Haar in der Suppe suchten, war für die Modedesignerin nervig.
38. Dass der Aktionär den Wald vor lauter Bäumen nicht sehen konnte, war nervig für die Geschäftsführerin.
39. Dass der Techniker versuchte, ihr die Katze im Sack zu verkaufen, war nervig für die Oma.
40. Dass die Reparatur des Wagens ein Schuss in den Ofen war, war nervig für den Mechaniker.
41. Dass eine Tasse Kaffee ihrem Gedächtnis immer auf die Sprünge helfen konnte, war für die Kellnerin angenehm.
42. Dass sie kurz vor der Wahl dem Gegner die Leviten las, war für die Kanzlerin wichtig.
43. Dass ihre Beliebtheit Schnee von gestern geworden ist, war traurig für die Köchin.
44. Dass ihre Patienten ohne Punkt und Komma redeten, war nervig für die Heilkundige.
45. Dass ihre Leberwurst unter aller Kanone schmeckte, war nervig für die Metzgerin.
46. Dass seine Gattin die Lunte nicht riecht, war wichtig für den Ehemann.
47. Dass ihre Kunden die Perlen vor die Säue warfen, war nervig für die Vermögensberaterin.
48. Dass sie dem Klempner nach der Fehlreparatur aufs Dach stieg, war wichtig für Karla.
49. Die Kletterin hatte das Gefühl, dass ihre Goldmedaille der höchste Berg in ihrem Leben war.
50. Dass der böse Winter endlich weggegangen war, war für die Medizinerin befreiend.
51. Dass die Sonne sie heute morgen sehr schallend angelacht hat, war angenehm für die Kauffrau.

52. Dass ihr Ehemann mit ihrer Schwester einen anderen Weg einschlagen wollte, war traurig für Katharina.
53. Die Bäckerin hat ihren Kompass hinsichtlich der Zukunft ihres Ladens verloren.
54. Maria wusste, dass die Dissertation zu schreiben eine steile und lange Reise ins Unbekannte sein würde.
55. Dass er mit der neuen Ernte einen Sechser gewürfelt hat, war schön für den Landwirt.
56. Dass seiner Beziehung das Benzin ausgegangen ist, war traurig für den Busfahrer.
57. Dass die Firma nicht mehr so schnell rennt wie früher, war nervig für die Managerin.
58. Dass ihre Argumente die Langeweile der Zuhörer durchsiebt haben, war schön für die Rednerin.
59. Dass die Kritik des Freundes im Kreisverkehr fuhr, war lustig für die Köchin.
60. Dass ihr Berufsleben in einer Sackgasse steckt, war traurig für die Jägerin.
61. Dass der Patient ihre Ratschläge mit Füßen trat, war traurig für die Physiotherapeutin.
62. Der Gärtner war zufrieden, sein Geschäft weiter wachsen und erblühen zu sehen.
63. Dass sie sich mit der Wahl ihres Geschäftspartners verzockt hat, war traurig für die Optikerin.
64. Dass ihre Mutter ihr wegen ihrer Noten den Krieg erklärt hatte, war amüsant für die Studentin.
65. Dass der Kommunist Tage brauchte, um die Ideen des Genossen herunterzuschlucken, war kein Zufall.
66. Dass sein Gast vor Wut gebrutzelt hat, war sehr lustig für den Showmaster.
67. Dass der Tod seines Angestellten einen Schlag ins Gesicht für seine Finanzen bedeutete, war für Anton traurig.
68. Die Chefin hatte es nie einfach, da Ihr Berufsweg immer kurvig und voller Steine war.
69. Die Gattin war hoch erfreut, als der Herzog den ganzen Raum mit seinen Ideen durchleuchtete.
70. Die Regisseurin glaubte, dass sich ihr Film, nach der letzten Kürzung, in der Notaufnahme befindet.
71. Als ihr Computer nach einem kurzen Leben gestorben ist, war die Informatikerin sehr enttäuscht.
72. Dass die Krebsdiagnose seine Lebenserwartung bis zum tiefsten Ozeangrund senken würde, war dem Seemann klar.
